# Proteomics: An Essential Tool to Study Plant-Specialized Metabolism

**DOI:** 10.3390/biom14121539

**Published:** 2024-11-30

**Authors:** María José Martínez-Esteso, Jaime Morante-Carriel, Antonio Samper-Herrero, Ascensión Martínez-Márquez, Susana Sellés-Marchart, Hugo Nájera, Roque Bru-Martínez

**Affiliations:** 1 Plant Proteomics and Functional Genomics Group, Department of Biochemistry and Molecular Biology and Soil and Agricultural Chemistry, Faculty of Science, University of Alicante, Carretera San Vicente del Raspeig s/n, 03690 San Vicente del Raspeig, Alicante, Spain; jaime.morante@ua.es (J.M.-C.); antonio.samper@ua.es (A.S.-H.); asun.martinez@ua.es (A.M.-M.); susana.selles@ua.es (S.S.-M.); roque.bru@ua.es (R.B.-M.); 2 Alicante Institute for Health and Biomedical Research (ISABIAL), 03010 Alicante, Spain; 3 Plant Biotechnology Group, Faculty of Forestry and Agricultural Sciences, Quevedo State Technical University, Av. Quito km 1 1/2 vía a Santo Domingo de los Tsachilas, Quevedo 120501, Ecuador; 4 Research Technical Facility, Proteomics and Genomics Division, University of Alicante, 03690 San Vicente del Raspeig, Alicante, Spain; 5 Departamento de Ciencias Naturales, Universidad Autónoma Metropolitana–Cuajimalpa, Av. Vasco de Quiroga 4871, Colonia Santa Fe Cuajimalpa, Alcaldía Cuajimalpa de Morelos, Mexico City 05348, Mexico; hnajera@cua.uam.mx; 6 Multidisciplinary Institute for the Study of the Environment (IMEM), University of Alicante, 03690 San Vicente del Raspeig, Alicante, Spain

**Keywords:** specialized plant metabolite, proteomics, secondary metabolism, mass spectrometry, natural products or compounds, biosynthesis

## Abstract

Plants are a valuable source of specialized metabolites that provide a plethora of therapeutic applications. They are natural defenses that plants use to adapt and respond to their changing environment. Decoding their biosynthetic pathways and understanding how specialized plant metabolites (SPMs) respond to biotic or abiotic stress will provide vital knowledge for plant biology research and its application for the future sustainable production of many SPMs of interest. Here, we focus on the proteomic approaches and strategies that help with the study of plant-specialized metabolism, including the: (i) discovery of key enzymes and the clarification of their biosynthetic pathways; (ii) study of the interconnection of both primary (providers of carbon and energy for SPM production) and specialized (secondary) metabolism; (iii) study of plant responses to biotic and abiotic stress; (iv) study of the regulatory mechanisms that direct their biosynthetic pathways. Proteomics, as exemplified in this review by the many studies performed to date, is a powerful tool that forms part of omics-driven research. The proteomes analysis provides an additional unique level of information, which is absent from any other omics studies. Thus, an integrative analysis, considered versus a single omics analysis, moves us more closely toward a closer interpretation of real cellular processes. Finally, this work highlights advanced proteomic technologies with immediate applications in the field.

## 1. Introduction

Plant secondary metabolites constitute a vast range of very chemically diverse compounds that plants have evolved to produce in response to a specific environmental stimulus, and also present a role in plant physiology. Specialized plant metabolite (SPM) is the preferred term, instead of plant secondary metabolite. It implies that this reservoir of plant compounds plays a specialized role, rather than a secondary one [1]. Unlike their primary counterparts, plant secondary metabolites do not appear to participate directly in plant growth and development. Primary metabolites are ubiquitous in the plant kingdom and perform essential metabolic roles for plants, such as respiration, photosynthesis, growth or reproduction. Secondary metabolites are often taxon- or species-specific compounds in plants and are not widespread. A prominent role of SPMs is the plant’s integral communication with its environment, including its response to biotic and abiotic stresses, and mediating interactions with other plants, insects and microbes [2]. Plant-specialized metabolism serves important ecological roles that help with plant adaptation and survival. Therefore, SPMs are used in this review, although other names for this type of compounds co-exist in the literature, such as “plant natural products”, “natural compounds” or “phytochemicals”.

Plants are sessile organisms that adapt to the environment and respond to pathogen attacks by a defensive mechanism that involves SPM biosynthesis. Under favorable conditions, it is known that plants direct their resources toward development and primary metabolism but prioritize defense-related compounds production in a stressful environment [3]. Thus, SPMs’ multiple biological roles include assisting plants in alleviating stresses caused by abiotic stresses, such as heat, drought, salinity and ultraviolet radiation [4]. They are also involved in attracting either symbiotic bacteria and arbuscular mycorrhizal fungi or pollinators. SPMs can act as defensive-specialized metabolites. Apart from defensive antimicrobial activities, some of these phytoalexins in plants present health-promoting effects. Of the many examples that can be found, numerous indole monoterpene alkaloids present pharmaceutical properties like anticancer vinblastine and vincristine, antimalarial quinine, antihypertensive ajmalicine or antiarrhythmic ajmaline [5]. Glucosinolates contribute to the antioxidant, cardioprotective and anticarcinogenic properties of Brassicaceae mustards [6]. Resveratrol, a phenolic-type stilbene compound, has anti-aging, anticancer, anti-inflammatory and antioxidant activities of interest for treating chronic diseases [7].

SPMs represent a valuable reservoir of natural compounds, which, given their unique chemical properties, present multiple pharmacological and biotechnological applications. For example, SPMs are used as agrichemicals, dyes, flavors, fragrances or medicines. Given the bioactive properties found in plenty of SPMs, they are sometimes referred to as plant bioactive compounds or added value products. Interest in SPMs has increased worldwide in the last 40 years. Modern medicine is based on knowledge built over centuries about how medicinal plants have been used to cure diseases. As recently reviewed by D’Amelia et al. (2021) [8], the origin of 70% of the 1562 approved drugs from 1981 to 2019 in the US is natural [9]. The WHO has reported that around 40% of pharmaceutical products today are plant-based, either because of their natural origin or because they are synthetically inspired in a natural product, including landmark drugs like aspirin and artemisinin and childhood cancer treatments like vinblastine and vincristine [10,11]. The growing demand for bioactive compounds imposes ecological pressure on natural resources. To avoid overexploiting our planet’s natural resources and to preserve its biodiversity, biotechnological solutions are proposed in the literature as reviewed elsewhere [8]. Hence, the application of biotechnological strategies can lead to sustainable SPMs production. One example is using plant cell culture as green biofactories to produce SPMs, according to the elicitation strategy. Successful applications of this strategy include the production of resveratrol in *Vitis* sp. [12], paclitaxel in *Taxus* sp [13], silymarin in *Silybum marianum* [14] and ginsenosides in *Panax gingseng,* along with the many more examples reviewed by Ramirez-Estrada et al. (2016) [15].

All the above statements indicate the pivotal need for advancing our knowledge in plant-specialized metabolism. Understanding how plants respond to biotic and abiotic stress or climate change is vital for future cultivation and breeding strategies. Plants use an estimated 15–25% of their proteome for specialized metabolic pathways, which remain largely uncharacterized [16,17]. The discovery of natural product biosynthesis pathways is still a major obstacle to know the full potential for engineering specialized metabolites in plants and microbes. The discovery of key enzymes for the biosynthesis of SPMs and to decode their biosynthetic pathways will lead to sustainable production by applying biotechnological strategies via metabolic engineering.

Here, we focus on the proteomics strategies and approaches that add value to the study of plant-specialized metabolism. We present examples of how proteomics has contributed to the field in the last 10 years and highlight emerging strategies and technologies for immediate application to study plant-specialized metabolism. Finally, we emphasize the importance of proteomics as part of an integrative omics approach to decode plant-specialized metabolism.

## 2. Major SPM Classes

More than 200,000 compounds of diverse chemical structures have been identified to date. It is estimated that plants can produce more than 1 million SPMs. The best studied SPMs can be classified into three major groups based on their chemical structure: phenolics, terpenes and nitrogen-containing compounds (a diverse group that can also contain sulfur). Other groups of SPMs include specialized fatty acids and carbohydrate derivatives [18,19]. Some SPMs are preformed defenses because they are synthesized in healthy tissues and stored either in an active form or as inactive precursors in cells, or generally in vacuoles or organelles in the most external plant layers, e.g., trichomes. Examples of preformed defenses are saponins, glucosinolates and benzoxazinoids [1]. Some others can be de novo synthesized in response to biotic or abiotic stress and are, in this case, collectively named phytoalexins [20].

### 2.1. Phenolic Compounds

Plant phenolics compounds are a large, extremely diversified class of SPMs that can differ in size and complexity. An aromatic ring that bears at least one hydroxyl group is the distinctive structural feature that characterizes phenolic compounds. They can be further subdivided into several subclasses depending on the number of phenol units or the presence of other functional groups. So, they can be broadly classified into two major groups: flavonoids and non-flavonoids.

**Figure 1:**
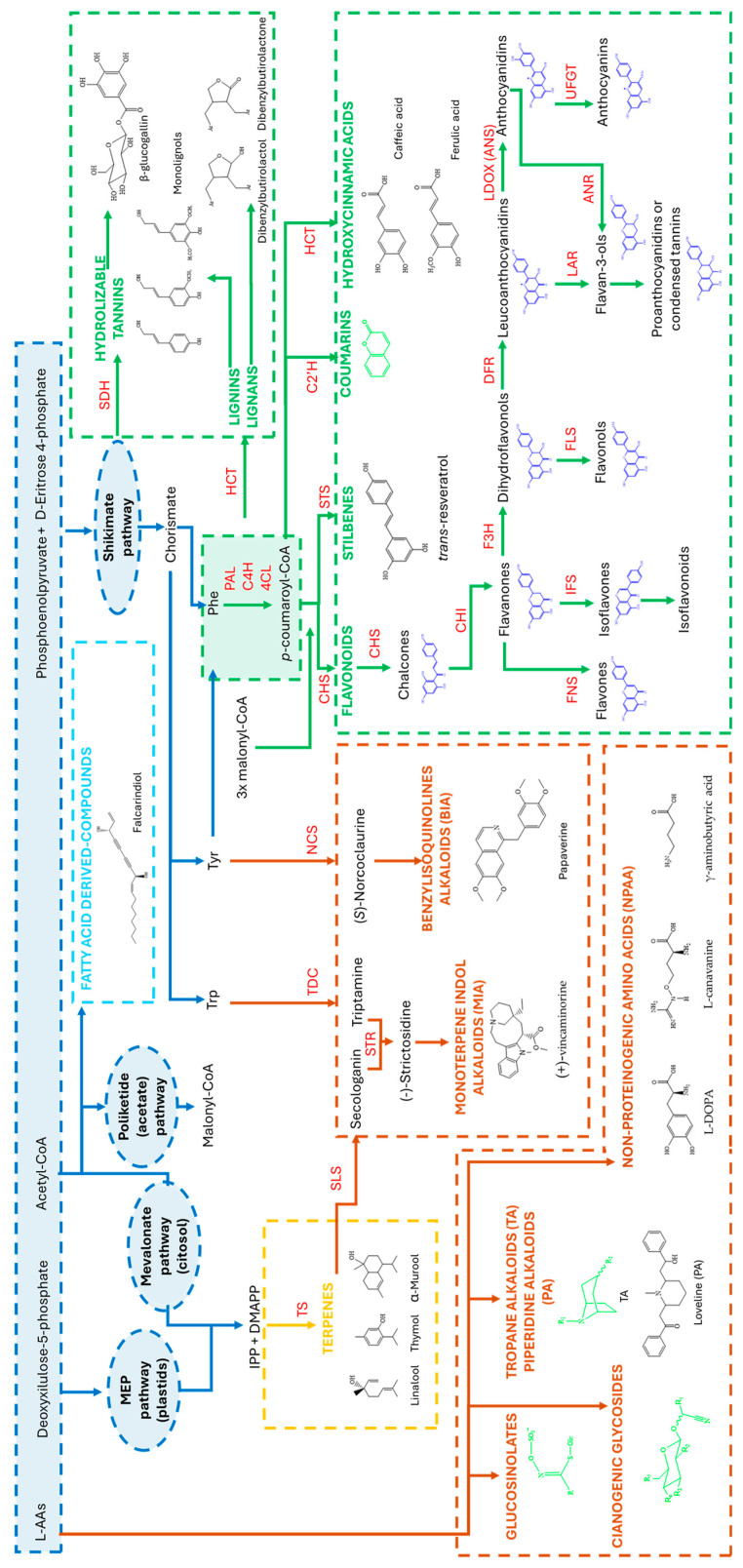
Schematic overview of the specialized (secondary) metabolism. The plant-specialized metabolic pathways related to phenolic compounds (in green), terpenes (in yellow), fatty acid-derived (in light blue) and N-containing compounds, glucosinolates, alkaloids, cyanogenic glucosides and non-proteinogenic amino acids (in orange) are shown. Dark blue denotes precursors and primary biosynthetic pathways. The structure of the representative examples of related compounds is represented in black. The basic skeleton structures for some groups of compounds are depicted in green, and those related to flavonoids in blue. Abbreviations: anthocyanidin 3-O-glucosyltransferase (UFGT); anthocyanidin reductase (ANR); chalcone synthase (CHS); chalcone isomerase (CHI); cinnamate 4-hydroxylase (C4H); coumarate CoA ligase (4CL); p-coumaroyl-CoA 2-hydroxylase (C2’H); dihydroflavonol 4-reductase (DFR); flavanone 3-hydroxylase (F3H); flavone synthase (FNS); flavonol synthase (FLS); hydroxycinnamoyl transferase (HCT); isoflavone synthase (IFS); leucoanthocyanidin reductase (LAR); leucoanthocyanidin dioxygenase/anthocyanidin synthase (LDOX/ANS); norcoclaurine synthase (NCS); phenylalanine ammonia lyase (PAL); secologanin synthase (SLS); shikimate dehydrogenase (SDH); stilbene synthase (STS); strictosidine synthase (STR); terpene synthase (TS); tryptophan decarboxylase (TDC).

Phenolic compounds are formed via phenylpropanoid and polyketide (acetate) pathways [21]. The phenylpropanoid pathway provides *p*-coumaroyl-CoA from phenylalanine (Phe) by three enzymatic reactions catalyzed successively by phenylalanine ammonia-lyase (PAL), cinnamate 4-hydroxilase (C4H) and 4-coumarate-CoA ligase (4CL). In most plant species, Phe is the precursor for the phenylpropanoid pathway, but some species can also use tyrosine (Tyr) as a substrate for PAL [22]. The acetate pathway provides malonyl-CoA for condensation with coumaroyl-CoA. Flavonoid biosynthesis starts with the condensation of *p*-coumaroyl-CoA with three malonyl-CoA molecules for chain elongation by chalcone synthase (CHS) to produce chalcones. The core flavonoid structure consists of three interconnected rings. Ring A is derived from *p*-coumaroyl-CoA, and ring B is formed by the condensation reactions involving malonyl-CoA by CHS. Although ring C is formed by the closure of the chalcone, a reaction catalyzed by a chalcone isomerase (CHI) leads to the formation of flavanones. Flavanones are located at an important branchpoint of the flavonoid pathway and serve as a biochemical precursor of other flavonoids. Modifications of the C ring produce different flavonoid classes, and modifications of rings A and B increase the compound diversification in each phenolic group. Different substitutions can appear in the basic unit (hydroxylation, glycosylation, methylation, prenylation or acylation) at distinct positions, and their number and combination, together with the stereochemistry, degree of polymerization and linkage between core units, produce a wide diversity of flavonoids [23]. Over 8000 different flavonoids have been reported and can be subdivided into flavones, flavonols, flavanols, flavanones, isoflavones, anthocyanidins, chalcones and proanthocyanidins [24]. Current knowledge about their biosynthetic pathways is reviewed elsewhere [21,23,24,25,26,27]. The general overview of the flavonoid biosynthetic pathway is described in Figure 1. The type, amount and localization of some flavonoids vary according to plant species and the developmental stage of tissues and may be modulated by environmental signals. Flavonoids protect plants from different biotic and abiotic stresses. Yet, given their antioxidant properties, flavonoids also play important roles in human health, as eating plant-derived foods can prevent degenerative diseases associated with oxidative stress [27].

Non flavonoids comprise phenolic acids, tannins, hydroxycinnamates, coumarins, stilbenes and lignin. *p*-Coumaroyl-CoA is the precursor for non-flavonoid phenolic compounds. In fact, *p*-coumaroyl-CoA resides at a critical position in plant-specialized metabolism. Alternatively, during an analog reaction to CHS, stilbene synthase (STS) catalyzes the synthesis of stilbene resveratrol (3,5,4′-trihydroxystilbene). Stilbenes occur in limited, but phylogenetically distant, plant families because STS, the key biosynthetic enzyme for stilbenes, is not ubiquitously distributed [28]. Of the 34 plant families in which resveratrol can be isolated, *Vitis* sp. is one of the few plant families with a high stilbenes content and is the main dietary source of resveratrol [28,29]. In a grapevine, the resveratrol skeleton can undergo different modifications catalyzed mainly by decorating enzymes, such as glycosyltransferases, methyltransferases and hydroxylases, along with peroxidases, which results in various resveratrol derivatives [30]. For lignin (G- and S-types) and lignan biosynthesis, hydroxycinnamoyl transferase catalyzes the first committed step of the pathway into the intermediates for their synthesis, the monolignols. Coumarin represents a major non-flavonoid class that derives from the phenylpropanoid pathway with diverse medical bioactivities [31]. Recent research provides new evidence for their biosynthetic pathway [32]. The biosynthetic process involves three distinct steps by three types of enzymes: *p*-coumaroyl CoA 2′-hydroxylase (C2’H), C-prenyltransferase (C-PT) and cyclases. Coumarins can be classified into simple coumarins, furano- and pyranocoumarins and other complex coumarins [33]

In plants, phenolic compounds are largely decorated by hydroxylation, methylation, glycosylation or other modifications that diversify SPM properties. *O*-methylation is a frequent modification of SPMs and has been reported to increase the antimicrobial activity of flavonoids [34] and stilbenoids [30]. Most methylation reactions are catalyzed by *O*-methyltransferases (OMTs). OMTs have limited substrate specificities [34] but some enzymes like caffeic acid *O*-methyltransferases (COMTs), are promiscuous and lead to difficulties in decoding biosynthetic pathways [35]. Another major modification is glycosylation, which is crucial for multiple characteristic chemical properties of SPMs in the cell, such as increased solubility, stability and the ability to accumulate in large quantities in vacuoles compared to the aglycone counterparts. They can also add different organoleptic properties to compounds. Many flavonoids and stilbenoids are glycosylated [30,36]. Prenylation exists in SPMs, as reported in stilbenes [30], coumarins [32] and flavonoids [37] due to its increasing bioactive properties [38]. The above-described modifications produced by hydroxylases, methyl- and prenyl-transferases not only serve to diversify SPMs, but also increase the arsenal defense of plants, and these reactions are also involved in their biosynthetic pathways. By way of example, caffeoyl-CoA-*O*-methyltransferase (CCoAOMT) and *C*-prenyl-transferase play a crucial role in the biosynthesis of lignin [39] and complex coumarins as furanocoumarins [40], respectively. Oligomerization is another common modification. Resveratrol dimers, and even higher-molecular-weight oligomers, possess superior bioactivities to those of monomers [29]. Most of the enzymes responsible for structural SPMs modifications are still unknown, which means that a whole suite of enzymes is still to be discovered.

### 2.2. Terpenoids

Terpenoids are the largest class of SPMs in plants and around 30,000 compounds have been identified to date. Terpenoids are also a structurally very diverse class of lipids, as described for phenolic compounds. They all derive from the isomeric five-carbon isopentane units, isopentenyl diphosphate (IPP) and dimethylallyl diphosphate (DMAPP). In plants, biological five-carbon isoprene units derive from two different routes: the mevalonate pathway (MVA) that occurs in the cytosol of cells, and alternatively via the methylerythritol 4-phosphate pathway (MEP), which occurs in plastids [41]. Both pathways are spatially separated and contribute differently to the formation of all terpenoids. The MVA pathway is used to produce sesquiterpenes, sterols and triterpenes, while the MEP pathway is employed in the production of monoterpenes, diterpenes, chlorophylls and carotenoids [41,42] and sesquiterpenes have also been reported [43,44]. Although each pathway seems to form different terpenoids, the exchange of isoprene intermediate units has been documented [45]. Thus, prenyl transferases condensate the five-carbon units, IPP and DMAPP, to form a series of longer prenyl diphosphates in multiples of five carbons. Terpene synthases (TSs) catalyze the conversion of prenyl diphosphates into terpene carbon skeletons to give rise to monoterpenes (C10), sesquiterpenes (C15), diterpenes (C20), triterpenes (C30) and tetraterpenes (C40). TS products are usually cyclic, often have multiple ring systems and comprise the primary representative of all the many diverse terpene skeletal types. After cyclization, the parent terpene skeleton undergoes further modifications, including oxidations, reductions, isomerization, conjugation, among other transformations. In diterpenoids, most diversity is generated by modifying enzymes, such as cytochrome P450 monooxygenases (CYP450), oxidoreductases, glycosyl and acyl transferases [46]. These modifications are poorly studied. Once again, a whole suite of enzymes is still to be discovered.

### 2.3. Nitrogen-Containing Compounds

#### 2.3.1. Alkaloids

Alkaloids are a large group of SPMs which possess very complex chemical structures that usually contain multiple stereogenic centers. More than 20,000 compounds have been identified to date. The very high diversity of alkaloids is based on their botanical, biochemical origin, but also on their structure and bioactive properties, and they are usually classified according to their precursor and biosynthetic pathway [47]. Nonetheless, taxonomic classification is also common to group alkaloids with a related structure in genus plant species; one such example is opium alkaloids [48]. In most cases, alkaloids are formed from L-amino acids, either alone or in combination with a terpenoid-type moiety. Others are also formed from nucleotides or polyamines, such as putrescine or cadaverine. The vast number of classes of alkaloids [47] have unique biosynthetic pathways that differ from most other types of specialized metabolites [49]. Several biosynthetic pathways have been elucidated and serve as an example of how alkaloids are synthesized [50]. However, the biosynthesis of most alkaloids remains undetermined. Considering the very high diversity and classes of alkaloids identified to date, only the most studied alkaloids in the revised literature are included in Figure 1.

Other comprehensive reviews exist of the known biosynthetic routes of alkaloids [47,49,50,51]. As an example, these include the compounds that are derivates from the aromatic amino acids tyrosine and tryptophan which are benzylisoquinolines (BIA) [52] and monoterpene indole alkaloids (MIA), respectively (Figure 1).

#### 2.3.2. Other Nitrogen-Containing SPMs

Glucosinolates are nitrogen-containing compounds and sulfur-rich SPMs. They constitute a small class of compounds that is largely restricted to the Brassicales order, including the genera of *Arabidopsis* and Cruciferae [53]. They are biologically active after hydrolysis by β-thioglucosidases, a myrosinase, to produce SPMs known as “mustard oil bombs” that are involved in plant defense. Glucosinolates differ in the side group depending on the amino acid that they derive from, including valine (Val), alanine (Ala), isoleucine (Ile), leucine (Leu), methionine (Met), Phe, Tyr and tryptophane (Trp).

Cyanogenic glucosides are α-hydroxynitriles that derive from five amino acids (Val, Ile, Leu, Phe and Tyr) and the non-protein amino acid cyclopentenyl-glycine, and they are conjugated with a glucose moiety. Cyanogenic glucosides are chemical defense molecules that protect plants from tissue disruption. They have the ability to release toxic hydrogen cyanide when hydrolyzed by β-glycosidases [54]. The core structures of cyanogenic glycosides may be further modified by hydroxylation. So, the diversity of cyanogenic glycosides is further extended by the structural diversity of the sugar moiety.

Natural endogenous peptides act as defensive compounds and present bioactive properties. Peptides are not usually considered to form part of SPMs. Although the biosynthetic process of peptides differs from that of other SPMs, they can be functionally classified as SPMs. Recently, their classification, structure and biosynthetic pathways have been comprehensively reviewed [55]. To date, over 1500 modified peptides have been isolated from plants, but most remain unknown. Complex natural peptide products are small molecules composed of amino acids linked by amide bonds that present diverse chemical structures. All plant natural peptides (PNPs) are synthesized in ribosomes composed of proteinogenic amino acids and are then further post-translationally modified. They are known as ribosomal-synthesized and post-translational-modified peptides (RiPPs). PNPs present a limited number of protein post-translational modifications (PTMs), including terminal modifications like N- or C- macrocyclizations or pyroglutamate formation. The crosslinking of amino acid side chains and other modifications, such as cystines, tyrosine sulfonation and proline hydroxylation, is frequent. Proteomics technology can assist in their identification and structure elucidation [56,57,58].

Non-proteinogenic amino acids (NPAA) can act as plant defense metabolites against biotic [59] and abiotic stress [60] beyond their already known physiological role as primary metabolites [61]. More than 700 NPAAs, not related to primary metabolism, have been reported from a wide variety of plants, including legumes, and are abundant in seeds [62]. NPAAs can originate from three main different pathways: some NPAAs arise from the structural modification of existing protein amino acids; others from the deviation of the pathways leading to protein amino acid; and a third group of NPAAs from novel routes. However, the biosynthesis of many NAAPs remains unknown. As an example, γ-aminobutyric acid (GABA) is a well-known NPAA produced in plants by glutamate decarboxylase with many different abiotic and biotic stresses [60] 

### 2.4. Other SPMs

Fatty acid-derived compounds are a group of well-studied molecules, such as the unusual fatty acids typically found in seed oils [18,63] or the derivative jasmonate-like oxylipins. However, an understudied, highly structurally diverse group of compounds from fatty acids with novel bioactive functionalities in plants have been recently reported, highlighting their interest for future research [19]. These include polyacetylenes, very long-chain taxon-specific compounds, and others with aromatic rings in their structure.

Other carbohydrate derivative compounds have been reported, such as the resin glycosides, a complex glucolipid with a defense role against herbivores found in plants of the Convolvulaceae family, which exhibit broad biological activities with pharmaceutical potential [18,64].

## 3. Tools to Study SPM Metabolism

The study of the metabolism of SPM by either deciphering their biosynthetic pathways or analyzing the effects that stress pressures inflict on SPM synthesis requires taking a comprehensive approach. Only their study at different hierarchical levels (metabolite, protein, RNA and gene, and their interaction), will take us closer to the real molecular scenario. Single-omics approaches provide us with valuable, but partial, information on the biological system under study. Thus, integrative omics-based approaches are now widely accepted as the main tool to study SPM metabolism, as reported thoroughly in previous reviews [4,17,65,66,67,68].

Genomic-based approaches, by means of genome mining, phylogenomic analysis or genome-wide association studies, have largely contributed to deciphering SPM biosynthetic pathways [65]. However, genomics alone is not sufficient to confidently identify SPM metabolic pathways. Transcriptomics has been the prevalent technology used to identify new enzymes and to elucidate biosynthetic pathways. The transcript analysis captures the co-expression of clustered or distal genes under tested conditions and at certain time points, and has guided the discovery of SPM biosynthetic pathways. The combination of transcriptomic with metabolic or genomic data has been essential for studying SPMs [1,69].

Measuring RNA transcription may not correlate to proteins abundance in a cell or tissue by giving only a deceptive link with proteins [70,71,72]. Transcriptomic and proteomic profiling studies have shown that the relation between mRNA and protein levels is complex and depends on cells’ state, and even on the organism in question. Some studies report a good mRNA/protein ratio in plants, especially where protein functions are more critical [73], and where strong induced transcription occurs [71]. Nevertheless, other studies report that a minor correlation between mRNA and protein in plants is observed when cells respond to stress [70,74,75]. Thus, protein abundance can only partially be explained by transcript abundance. Each step of the gene expression is precisely regulated, starting from transcription control, going through to mRNA posttranscriptional processing and its decay, translational and posttranslational regulation, and ending up with the protein degradation rate. It seems unlikely that a correlation exists between each mature protein or proteoform to arise from the expression process for their origin gene and their corresponding mRNA [72].

Furthermore, proteins define the cell’s functional state and determine its phenotypes. Mature and functionally active proteins are the effector molecules in the cell. Their activity can depend on posttranslational modification and/or proteolysis maturation, and their correct localization in the cell. The proteome is the closest molecular dimension in the cell related to the metabolome. Therefore, it is essential to complement transcriptomic and metabolomic data with the direct measurement of protein abundance if possible [76]. Indeed, the understanding of the pathways involved in SPM metabolism, and their regulation, requires investigating the responsible enzymes, transporters and transcription factors that share the common feature of being proteins.

## 4. The Proteomics Toolbox

Proteomics is the characterization of proteomes, including the abundance of their protein constituents, of a cell, tissue or organism. To detect those proteins of interest in plant-specialized metabolic pathways, which may well be the case of many regulatory proteins or transcription factors, a characterization of the deep proteome is a must. This is to avoid the list of catalogued proteins consisting mostly of abundant proteins, referred to as those of a medium-high copy number, and to gain access to the least abundant proteins. Present-day technology, performed by advanced mass spectrometry (MS)-based proteomics, enables the analysis of complete proteomes [77,78], which means that the identification of a representative of all the encoded proteins of proteomes is potentially possible. Accordingly, MS may not be a major obstacle during the process [79]. Thus, for the comprehensive characterization of a proteome to be successful, sample preparation is a fundamental step in the proteomic workflow. To study SPMs, it is very important to consider the target proteome in the plant to be analyzed. Different strategies can be followed to enrich the desired metabolic pathways by selecting the suitable plant material regarding: (i) the plant part to be analyzed because some biosynthetic pathways or part of them are tissue-, organ- or cell-specific [49]; (ii) the subcellular compartment [80,81]; (iii) the age or developmental stage [82]; (iv) the specific environmental conditions [83]. Elicitation is a powerful strategy to trigger or enhance SPM biosynthesis and, consequently, for the increase in the expression of key enzymes and the activation of responsible metabolic pathways [84,85,86,87,88,89,90,91,92]. Mutants for a gene of interest can be generated for a characterization of the resulting phenotype. The production of a plant with either a ‘gain of function’ or ‘loss of function’ phenotype may help to study SPM pathways. Regarding sample preparation, protein extraction is not a universal protocol and needs to be specifically optimized for the successful recovery of the complete repertoire of proteins in cells, tissues, secretions or organs. Advanced sample preparation for high-throughput plant proteome characterization was analyzed in previous research, which could be beneficial for its application for difficult plant samples or dealing with low protein content as occurs in the study of plant-specialized metabolism [93,94]

**Figure 2:**
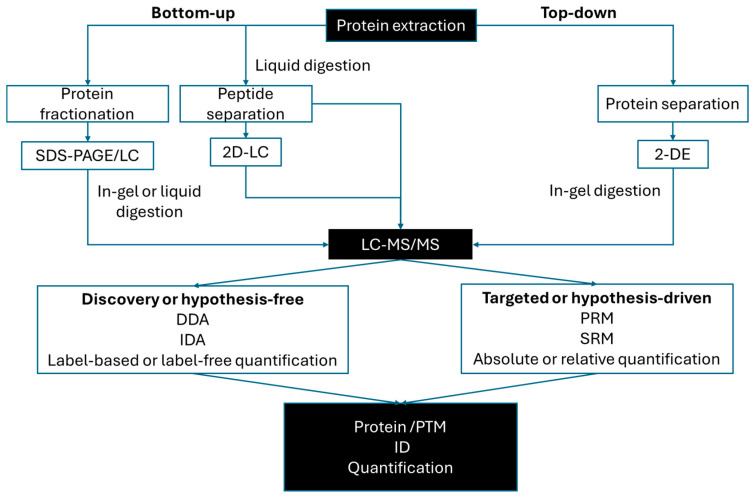
Proteomic approaches and strategies typically applied to analyze plant proteomes. Here, the top-down approach follows a comprehensive-centric definition that refers to protein separation by 2-DE prior to MS, while bottom-up applies to sample separation at the peptide level prior to the peptide analysis by MS. When dealing with complex samples, they can be extensively fractionated before the protein digestion step by either SDS-PAGE [113,114] or HPLC. Wider proteome coverage can be achieved by 2D protein separation, where orthogonal separation is applied in combination with the second peptide separation by the standard RP-HPLC, coupled online to MS. The additional separation step can consist of up-front chromatography based on high pH chromatography or strong cation exchange (SCX), which are among the most widely used techniques. Then, the digested proteins from gel slices or chromatographic fractions are analyzed by LC-MS/MS. On the one hand, if the goal is to identify new proteins of interest, a discovery or hypothesis-free approach is undertaken. In this case, MS data acquisition can be done by either DDA or IDA. Protein abundance can be determined simultaneously to protein identification by either label-free or label-based quantification methods, of which iTRAQ/TMT is the most widely used. On the other hand, having identified proteins of interest, a targeted or hypothesis-driven approach can be applied with different goals, to do the following: accurately quantify a protein by absolute quantification; validate protein abundances obtained from a discovery-based approach; selectively analyze a metabolic pathway under different conditions. In this case, MS data acquisition can be done by either an SRM or a PRM analysis.

The characterization of proteomes without previous knowledge about the proteins of interest is known in proteomics as hypothesis-free or discovery studies. They mainly rely on two main approaches: top-down and bottom-up (Figure 2). The top-down approach refers to the characterization of proteoforms [95] following a protein-centric approach. Although the top-down approach has been widely applied to identify intact proteins by an MS analysis, also referred as top-down MS analysis [96], the top-down concept is also used for the characterization of proteomes based on their separation at the protein level. In this case, the top-down approach can be understood as a comprehensive-centric definition of the proteome analysis [72]. As described here, up-front high resolution is achieved using classic two-dimensional electrophoresis (2-DE) and then proteins are identified following the in-gel-digestion of the resolved proteins and the analysis of the resulting peptides by liquid chromatography coupled to mass spectrometry (LC-MS). In contrast, in bottom-up approaches, also known as the shotgun analysis [97], the peptides released from the complete digestion of the protein extract by a sequence-specific enzyme like trypsin are resolved and analyzed by MS [79]. So, in the bottom-up approach, the MS analysis very much depends on a highly resolved peptide complex mixture using reverse-phase liquid chromatography (RP-HPLC) coupled to MS. Both top-down and bottom-up approaches are considered to be complementary, but offer clearly different advantages. Until a few years ago, 2-DE was the classic, extensively used technique, until the shotgun analysis exceeded proteome coverage with a faster workflow. However, 2-DE still remains a powerful technique to identify different proteoforms that derive from either different isoforms or PTMs [72]. Alternatively, the proteome analysis can follow a hypothesis-driven strategy if proteins of interest are known, following a targeted proteomics approach [98]. In this strategy, only a predetermined set of peptides is selectively monitored very sensitively and specifically, and it is even comparable to the immune-based methodology [99].

Irrespective of the applied approach (i.e., bottom-up or top-down, hypothesis-free or driven), the analysis ends with the peptide analysis by LC-MS/MS (Figure 2). This makes MS the core analytical technique for the analysis of the protein dimension of biological systems. Three main data acquisition methods are currently employed, which are data-dependent acquisition (DDA), data-independent acquisition (IDA) and targeted acquisition, in either the selected reaction monitoring (SRM) or parallel reaction monitoring (PRM) modes [79,100]. The preferred method for discovery studies is DDA, where the most intense precursors are selected for their fragmentation for MS/MS. DDA is a very sensitive method used to characterize proteomes, and successfully allows the comprehensive identification of the proteomes for cannabis, sweet cherry and pear with 13,850, 7584 and 17,983 proteins, respectively [101,102,103] However, the main disadvantage that is presented by DDA is its bias for abundant proteins, which affects the accuracy and reproducibility of the data. In IDA, all the co-eluted precursors are fragmented in a multiplexed fashion [104]. In theory, a profounder proteome analysis is achieved by the IDA analysis due to its capability to detect low-abundance proteins, but this MS method requires a more intensive bioinformatic analysis of the acquired spectra to deconvolute each peptide from the IDA data. A popular variant of this MS method is SWATH-MS, in which the complexity of IDA data is decreased by systematically acquiring the entire fragmented ions present in sequential wide precursor isolation mass windows (i.e., 25 *m*/*z*). In recent years, the use of IDA and SWATH acquisition has increased for the analysis of plant proteomes [105,106,107,108]. DIA has proven effective in the case of low sample amounts [109] which indicates that it is a powerful tool for the analysis of certain samples as single-cell proteome analysis or subcellular fractions which yield low protein amounts [110]. Many possible instrument configurations dominate discovery proteomics [104,111]. In the targeted approach, triple quadrupole instruments dominate SRM data acquisition. Alternatively, PRM acquisition at a high resolution, such as orbitraps, can also perform a targeted analysis by using the entire MS/MS spectrum, rather than predefined transitions based on the *m*/*z* pair per precursor and its specific ion product [112].

The proteome is a highly dynamic entity. Thus, to gain insight into any biological system, a proteomic analysis must provide accurate quantitative information of the identified proteins. Quantitative proteomics, mainly by MS-based approaches, can deliver the protein abundances or protein accumulation levels of the different proteoforms in analyzed samples [115,116]. These protein abundances can be measured in an absolute or relative fashion. Absolute quantification reports the molar abundance of proteins and, to do so, a peptide- or protein-specific calibrant, generally an isotopically labeled standard [117], is required. However, other strategies based on non-labeled standards have been developed [118]. The main limitations of absolute quantification are its cost, that it is a laborious and time-consuming approach and that it only allows a limited number of proteins to quantify in one experiment. In the context of systems biology research, a semi-absolute quantification was developed for a more realistic and practical application of large-scale proteome quantification [119]. Providing molar quantities of the proteins determines their stoichiometry in protein complexes, pathways or networks which could provide valuable information for the study of plant-specialized metabolism. However, only scarce studies are available for the analysis of plant proteomes [120,121]. Relative quantification refers to the relative protein abundances captured in the comparison between different samples, which is the most used strategy when quantifying proteomes on a large scale. Two main strategies can be applied, either label-free or label-based methods. The various available methodologies can be considered complementary for offering different advantages or disadvantages. Label-free quantification (LFQ) is widely used because it is cost-effective, it requires minimal sample manipulation, and it allows us to quantify an unlimited number of samples. The main drawback of this type of quantification is its poor measurement precision, typically having a 20% coefficient of variation (CV) [116], mainly due to technical variability during sample preparation. Existing post-acquisition treatments of the MS data perform an alignment of peptide signals, which improves the typical missing values of DDA and also correct differences due to sample prefractionation. [122]. The label-based methodology refers to the isotopically stable chemical modifications of peptides by adding a label or a tag, which produces a measurable and distinctive change in the *m*/*z* value of modified peptides. Multiple label-based methods are available, and they all offer the common main advantage of multiplexed analysis by minimizing experimental variability, because parts of the sample procedure and acquisition during an MS analysis are applied simultaneously to all the samples in the experiment. Multiplexed proteomics experiments report very high reproducibility, with CVs of about 5 %, and very few peptides with CVs above 10 % [116]. Each label-based method has its own principle, sample preparation, MS acquisition and postacquisition analysis. The main disadvantage is the limited number of samples to quantify, because these methods are commercially available and provide a limited number of tags. Isobaric methods, such as the tandem mass tag (TMT) and the isobaric tag for relative and absolute quantitation (iTRAQ) are the most frequently used label-based methods in plant quantitative proteomics [123,124]. Briefly, labels of the same mass but different isotopic compositions are added to peptides, which are then combined for their analysis. In this case, quantification is based on the information within the MS/MS spectra obtained after peptides fragmentation and the release of the tag with a distinctive *m*/*z* because of its different isotopic composition. Other label-based methods are available in which peptides are chemically modified, as reviewed elsewhere [116,125]. Comparison among different quantitative strategies showed that isobaric tags present comparable reproducibility to DIA acquisition methods, and present a higher quantitative accuracy compared to label-free quantification [126,127]. Although label-based approaches are still the gold standard for MS-based quantification, in recent years label-free approaches have become the workhorse of quantitative proteomics, for their simpler experimental design, sample preparation methods and the possibility of an unlimited number of samples to compare in the same experiment [128].

MS-based proteomics allows for the global and system-wide characterization of protein modifications, because PTMs cause a defined mass shift that can be measured with the resolution of a single amino acid to provide high accuracy by MS of the PTM assignment. Considering the substoichiometric nature of PTM modifications, modified peptides are very difficult to detect, and an enrichment strategy is needed to overcome the issue of their lower abundance compared to the non-modified version of the peptide. The chemical properties of the PTM group are used to enrich modified peptides. Phosphorylation is the most studied PTM, and the commonly used enrichment strategy is based on metal chelation [129]. The use of titanium dioxide (TiO_2_) material is routinely applied to capture phosphopeptides, which are then analyzed following one of the abovementioned workflows in proteomics. Another methodology has been developed for the sequential enrichment of PTMs by making the simultaneous analysis of phosphorylated and N-glycosylated proteins possible [130]. Proteomics is used to identify other modified peptides in plants that bear the following PTM group, such as ubiquitination, SUMOylation, acetylation, and other less studied examples, such as crotonylation and 2-hydroxyisobutyrylation [131,132,133,134,135,136,137]. In recent years, the number of research works about PTMs in plant species has steadily increased [123,124]. Therefore, several resources have been released to assist the study and interpretation of PTMs in plants, including an integrative quantitative PTM database [138] based on curated published data, a functional analysis tool for post-translational modifications (FAT-PTM) that supports 49,000 PTM sites identified in large-scale proteomic surveys of the model organism *Arabidopsis thaliana* [139] and the PTM viewer, an online tool that provides an overview of plant PTMs with 112,000 modified peptides and the tools to assess it [140].

Proteomics offers a technological solution in which MS is the core analytical approach that serves to detect the proteoforms contained in the proteome, by studying their structure and abundance to capture the dynamic properties of the proteome in terms of space and time. Therefore, proteomics is an essential tool to understand biosynthetic pathways, their regulation, and how they adapt to different external stimuli or stress to produce SPMs.

## 5. Proteomic Applications in the Study of SPMs

### 5.1. Deciphering Biosynthetic Pathways and Identifying Key Enzymes

**Figure 3:**
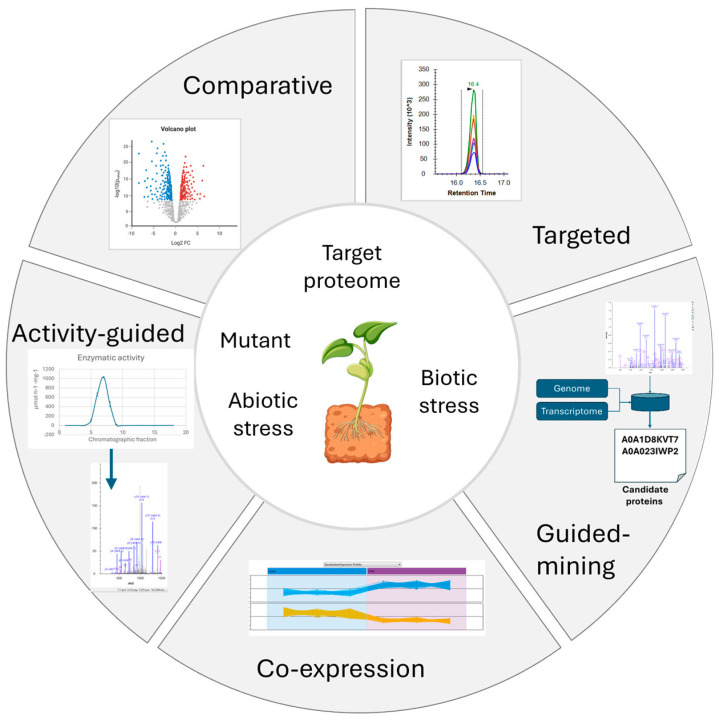
Current proteomic approaches applied for the study of plant-specialized metabolism. The plant material and the sample preparation procedure are critical for gaining access to target-specialized metabolic pathways and, therefore, the target proteome. So, different strategies can be followed to enrich the desired metabolic pathways regarding the selection of suitable plant material (tissue, organ, cell-type, subcellular compartments or development stage) and the conditions that enhance, annul or trigger the production of the SPM under study (mutant generation, elicitation or specific environmental conditions). The successfully applied proteomic approaches were comparative or differential proteomics, co-expression analysis, activity-guided proteomics, targeted proteomics and proteomics-guided mining approaches.

Given the complexity of the study of SPMs, different approaches are needed to advance our knowledge of the biochemical pathways responsible for SPM biosynthesis. Although candidates can be identified with transcriptomic and genomic approaches, as discussed above, the main challenge is to prioritize the candidate genes to be tested, due to the large amount of data generated. Proteomics can effectively assist in identifying a list of candidate proteins for the later characterization of their function. In this section, we report the proteomic approaches that have been applied in recent research to discover candidate enzymes and to clarify the biosynthetic pathways that lead to SPM synthesis to overcome difficulties in the study of SPMs. Figure 3 schematizes the proteomic approaches herein described.

#### 5.1.1. Comparative Proteomics

A classic approach in proteomics is the comparison of different conditions through a relative differential quantification proteomic pipeline. In a typical comparative protein expression study, multiple LC-MS runs are compared to identify the differentially abundant proteins between distinct biological groups and conditions. Advanced proteomics methods result in a powerful strategy to compare plant organs or cell types, and to reveal the location where pathways, or part of them, occur. SPMs are deposited in specific tissues or specialized organs. Furthermore, not all metabolites are produced in the place where they are stored. For example, alkaloid biosynthesis is rather complex, and a transcript and its coded enzyme may be in different plant compartments. BIA biosynthesis and accumulation in opium poppy involve three phloem cell types and implicate the translocation of key pathway intermediates between sieve elements and laticifers. Shotgun proteomics in complementarity with immunofluorescence labeling resolves the cellular localization of morphine biosynthesis in opium poppy [141]. The three enzymes that operate in the late morphine and noscapine biosynthetic pathways from thebaine to morphine, thebaine 6-*O*-demethylase (T6ODM), codeinone reductase (COR), and codeine *O*-demethylase (CODM) have been up-regulated in latex compared to the stem. Eight proteins acting upstream of thebaine synthesis have been restricted to the stem, which indicates that earlier morphine pathway steps occur separately from laticifers, in agreement with previous research findings. Previous research also points out two models for BIA biosynthesis in poppy due to discrepancies in immunolocalization and focuses on the immunofluorescence labeling of tissue sections using antibodies raised against recombinant responsible enzymes. However, shotgun proteomics evidence an alternative method for determining the localization of BIA enzymes compared to immunolocalization [141,142]. In another study, organ-specific proteomics has been performed in *Morus alba* to compare the protein profiles of leaves, branches, and roots. In this case, the accumulation of two proteins involved in the flavonoid biosynthetic pathway, CHI and flavonoid 3′,5′-hydroxylase, proved specific for the root. Proteomic data, together with a higher content for total flavonoids, and specifically for mulberroside A, kuwanone H, chalcomoracin, and morusin, compared to branches and leaves, suggest that the main organ in which flavonoid biosynthesis occurs is the roots [143].

In addition to the comparative profiling of proteins in cells, tissues and organs, a powerful strategy to study SPM metabolism is the comparison of biosynthetic mutant plants, because it could potentially reveal unexpected metabolic connections between pathways. The strategy, applied for the metabolome profiling of gene-specific mutants in a biosynthetic pathway, has revealed new evidence for the connection between dependent pathways for the critical position that *p*-coumaroyl-CoA occupies in the SPM metabolism of *A. thaliana* [144]. Two well-characterized mutants, transparent testa4 (tt4) and transparent testa5 (tt5), showed deficiency in CHS, the entry point to the flavonoid pathway, and CHI, the enzyme that produces flavanone naringenin and act after CHS (Figure 1), respectively. The differential metabolome profile for *tt4*, *tt5* and the wild type (WT) of plant seeds lead to the accumulation of precursors, which increases the formation of various naringenin chalcone derivatives for *tt5* and the phenolic choline esters for *tt4*. Redirection of *p*-coumaroyl-CoA to sinapate-derived metabolite biosynthesis, phenylpropanoid-type compounds, has been observed. An unexpected possible connection between glucosinolate and phenylpropanoid biosynthesis was suggested by the authors and later demonstrated in other studies [145]. The exemplified approach has been classically applied to infer the function of a gene in which the mutants for the gene of interest are generated and subjected to a phenotypic analysis by a metabolic analysis [146]. Nevertheless, the study of the comparative proteomics of the mutant vs. WT for the study of SPM metabolism is scarce in the revised literature to our knowledge. Mackon et al. (2024) [147] recently compared the black grain rice WT phenotype to a knockout line of *OsGSTU34*, a Bz2-like anthocyanin-related glutathione transferase transporter, which resulted in a complete loss of pigmentation in several parts of caryopsis, namely flowers, leaves, shoots, and roots. Comparative proteomics by TMT labeling has detected 1175 deregulated proteins. The 408 down-regulated ones included anthocyanin-related enzymes (PAL, DFR, F3′H, Glycosyltransferare (GYT), CHS, CHI, CL2), together with the *OsGSTU34*-related protein and directly anthocyanin-related enzymes, such as LDOX/ANS. However, the most important anthocyanin biosynthetic enzymes, DFR, GYT and LDOX/ANS, were not among the top 50 down-regulated proteins, with qRT-PCR showing that the expression of PAL, DFR, and ANS was the same in both the WT and mutant. The authors pointed out that the obtained results suggested that only the accumulation of anthocyanins was affected, but not their synthesis. Other examples related to the study of plant development exist. The effect of stress on root development in *A. thaliana* shows the effective application of shotgun comparative proteomics to study the signaling pathways that respond to diverse external stimuli at the protein level. The comparison of two mutants for two mitogen-activated protein kinase- (MAPK) encoding genes, mpk4 and mpk6, has allowed new proteins to be identified as putative MPK4 and MPK6 phosphorylation targets [148].

Comparative proteomics is a powerful approach to study SPM pathways in elicited plant cell cultures where specific metabolic pathways involved in the synthesis of SPM of interest are strongly induced. Upon elicitation, the identification of the implicated enzymes is favored compared to the analysis of plant tissues, organs or cells [149]. By way of example, resveratrol is a known stilbene produced in grapevine in association with resistance to fungi and is primarily found in grape berry skin. The enzyme responsible for its biosynthesis, STS, is not identified in either fruit [150,151] or skin [152] but several isoenzymes have been identified in elicited cell cultures [153,154]. In another example, a comparative shotgun analysis was performed during *Lithospermum erythrorhizon* cell growth in two different media and illumination regimes, light or dark, to represent shikonin-producing and non-producing cell culture phenotypes [155]. *L. erythrorhizon* cell cultures prove to be a suitable system for the study of shikonin biosynthesis, because they produce large amounts of shikonin derivatives and their production can be negatively regulated by light and a modified Murashige-Skoog’s (MS) medium for cell growth. The proteomic analysis revealed that proteins accumulated specifically in the shikonin-producing phenotype versus the non-producing cell cultures. Accordingly, shikitonin derivatives shikonin, acetylshikonin, deoxyshikonin, β,β′-dimethylacrylshikonin and α-methyl-n-butylshikonin were identified. The known enzymes involved in shikitonin biosynthesis clustered with the candidate enzymes responsible for the later unknown enzymatic steps. Based on the proteomic analysis, half of the later unknown pathway was proposed, including the biosynthetic pathway for naphthalene ring formation in shikonin and the synthesis of other shikonin derivatives. Candidates included an ortholog of cannabidiolic acid synthase, which cyclizes the geranyl side chain of cannabigerolic acid in *Cannabis sativa*; three acetyltransferases, all of which are orthologs of deacetylvindoline *O*-acetyltransferase in *Catharanthus roseus*; and polyphenol oxidases. A comparative proteomics analysis also proved to be successful to study other biosynthetic pathways in elicited cell cultures as reported in the literature: resveratrol and its derivates in *Vitis vinifera* [153,154] and *M. alba* [156]; and quinonemethide triterpenes in *Maytenus ilicifolia* [157] or terpenoid indole alkaloids in *Madagascar periwinkle* [158].

#### 5.1.2. Co-Expression Analysis

The expression of the genes of a particular SPM pathway may be highly correlated with one another because of coordinated transcriptional regulation. A co-expression analysis using transcriptomic data is one of the most powerful approaches for candidate biosynthetic gene identification in non-model plants [17,65]. The weighted gene co-expression network analysis (WGCNA) algorithm is commonly used for constructing a gene co-expression network. In gene co-expression networks, those genes with common expression profiles in different samples are present in the same gene network, and the co-expression relation between genes is generally determined by the expression correlation coefficient between those genes [65]. Proteomics lacks sensitivity compared to a transcriptomics analysis, as it usually presents a bias toward the most abundant proteins and tends to display a limitation in detecting low-abundance proteins. Despite transcriptomics being more sensitive, the transcriptome cannot reflect the phenotype because, as previously discussed, it fails to detect post-transcriptional regulatory processes, like PTMs. However, an integrated analysis of the transcriptome and proteome provides complementary information to draw more informative conclusions. The co-expression of differential accumulated proteins by a proteomic analysis increases the potential of the co-expression analysis of genes to decode unknown parts of biosynthetic SPM pathways. In addition to the WGCNA analysis, other multivariate analyses, based on correlation analysis and hierarchical clustering, have been used to analyze quantitative proteomic data [159]. A global data-independent acquisition proteomics analysis of four tobacco varieties with distinct nicotine contents has been performed. It identified 6018 proteins and, by using the WGCNA algorithm, 32 co-expression modules from root tip samples were detected. An expression module closely related to nicotine content contained the proteins mainly involved in the synthesis and metabolism of nicotine precursors, such as arginine, ornithine, aspartate, proline and glutathione. The increased levels of these precursors led to the synthesis and accumulation of nicotine in plants. More importantly, these proteins regulate nicotine synthesis by affecting the formation of putrescine, which is the core intermediate product in nicotine anabolism [160]. In another study, a shotgun analysis to compare laticifers of three poppy chemotypes revealed that several major latex protein-/pathogenesis-related 10 (MLP/PR10) family members with a similar protein abundance profile, which clustered together with the upstream biosynthetic enzymes of morphine biosynthesis providing the transcriptomic analysis with further evidence. The later screening of the candidate proteins for neopinone isomerase activity was performed. It detected 1 protein out of the 19 candidates responsible for the reaction. A novel MLP/PR10 protein, named neopinone isomerase, was revealed to be responsible for the isomerization of neopinone to codeinone and for the analog reaction of neomorphinone to morphinone, both of which were thought to be spontaneous until then [161].

A co-expression time-series analysis could reveal a deeper level of interaction [65]. Gene expressions change over time in response to environmental triggers and are regulated by multiple transcription factors (TFs) that work synergistically to cause a transcriptional cascade. A time-based study design can capture protein abundance fluctuations at different time points and under multiple conditions to increase the effectiveness of differential co-expression analyses. A hypothesis-free gel-based quantitative proteomic experiment by the DIGE technique of a temporal series of elicited grapevine cell cultures has been performed to explore the expression profiles of the known enzymes involved in *trans*-resveratrol (*t*-R) biosynthesis and other co-expressing proteins that are potentially involved in such a cell response. A correlation between two tau class glutathione-S-transferases (GSTs) with several STS and PAL isoforms with the *t*-R metabolite accumulation profile provided the basis for its selection as a candidate protein part of the machinery for transporting t-R to the extracellular medium. A functional analysis of VvGSTU10 promoted *t*-R transport to the extracellular medium in stably overexpressing grapevine cells. Therefore, the DIGE technique proved successful for detecting a candidate protein, GST-tau, involved in the extracellular accumulation of resveratrol in grapevine cell cultures elicited with methyl jasmonate (MeJA) and methyl-β-cyclodextrin (MBCD) [153]. In a later publication, the authors identified a candidate transporter, ABC- transporter B family member 15, by label-free proteomics of the tonoplast and plasma membrane fractions of elicited grapevine cell suspensions. The functional characterization of the transporter provided solid evidence for its involvement in *t*-R transport to the extracellular medium [162].

In addition to the temporal co-expression pattern, a proteomic analysis can also provide the spatial dimension. A quantitative proteomic atlas of 24 tissues in pear led to the identification of 17,953 proteins [103]. The protein-level evidence obtained by MS accounted for 42% of the predicted protein-coding genes in the pear genome. Based on the co-expression modules identified by the WGCNA of the proteome, the proteins involved in lignin biosynthesis, including CCoAOMT, HCT, CCR, C4L, F5H (ferulate/coniferaldehyde 5-hydroxylase) and LAC (laccase), were clustered together. A gene regulatory network to explore the internal connections between important regulators using proteome data from the same cluster showed several transcription factors/regulators, such as B3, homeobox, MADS domain proteins, as well as receptor-like kinases (RLKs), such as RLK-Pelle_LRR-III and RLKPelle_CrRLK1L-1, which were closely linked with lignin-related genes and may be involved in stone cell formation in pears. These results suggest that the key genes that control important traits can be explored by proteomic strategies.

#### 5.1.3. Activity-Guided Proteomics Approach

The power of proteomics technologies can assist in finding candidate enzymes by catalyzing the reaction of interest. When dealing with SPM metabolism, the existence of many enzymes capable of catalyzing similar reactions in their genomes is frequent and finding the responsible enzyme in such situations may be very difficult. Therefore, activity-guided approaches have been successfully applied to discover any unknown enzymes responsible for SPM synthesis, as recently reviewed [17]. To exemplify this approach, the study performed by Chen et al. (2018) showed the discovery of a key enzyme involved in the last thebaine biosynthesis step in opium poppy [163]. Thebaine is a product of the tyrosine-derived BIA pathway (Figure 1). Thebaine synthase (THS) synthesizes thebaine from (7S)-salutaridinol 7-*O*-acetate at the expense of the competing formation of unstable hydroxylated by-products. That reaction was a missing component of the biosynthetic pathway because the fact that the reaction spontaneously occurred was accepted [51]. THS was the protein Bet v1, an MLP member of the PR-10 family protein, which possesses unexpected enzymatic activity in SPM metabolism that renders its gene isolation via a homology-based reverse genetics approach unfeasible. These authors fractionated the protein extracted from latex in sequential purification steps, including hydrophobic interaction, ionic interaction and size exclusion chromatography coupled to the specific enzymatic assay. The partially purified and enriched fractions in the last SEC chromatography were subjected to SDS-PAGE, and the digested prominent bands were analyzed by LC-MS/MS proteomics. They also showed a substantial increase (>20-fold) in thebaine titers in genetically engineered yeast, which included the THS enzyme, and strongly impacted commercial opiate alkaloid synthesis [163]. In another study, Rhem et al. (2019) [164] investigated the N-terminal proteolytic cleavage processing of a cyclotide precursor, a type of PNP that is of particular interest for the pharmaceutical and agricultural industry, in *Nicotiana benthamiana*. They applied an activity-guided fractionation approach coupled to MS, which allowed them to identify a papain-like cysteine protease, kalatase A, responsible for the post-translational modification of the cyclotide precursor of cyclotide kalata B1 [164]. Martínez-Márquez et al., 2024 searched for enzymes that could catalyze the formation of piceatannol, a hydroxylated derivative of resveratrol, in grapevine cell cultures. For this purpose, three types of hydroxylation reactions were considered: NADPH-dependent cytochrome P450 hydroxylase; 2-oxoglutarate-dependent dioxygenase; cofactor-independent ortho-hydroxylation-like polyphenol oxidase (PPO) cresolase activity. However, it was demonstrated that PPO cresolase activity was the most probable route of piceatannol biosynthesis after the partial purification of the soluble fraction by precipitation with ammonium sulfate, dialysis and ion exchange chromatography due to marked PPO activity in certain chromatographic fractions, where the presence of the PPO protein was confirmed by Western blot and LC-MS/MS [165]. The search for the responsible enzyme for a key step in the biosynthetic pathway of a natural product can also be exemplified from organisms other than plants. By applying an enzymatic-driven approach, NADPH/FAD-dependent monooxygenase, VibO, was identified as the key enzyme for crucial oxygenation on the phenol ring needed to generate the oxepin-2-one structure of 1,5-seco-vibralactone, a natural product isolated from mycelial cultures of the mushroom *Boreostereum vibrans* [166].

#### 5.1.4. Proteomics-Guided Mining Approach

A proteomic analysis can provide evidence at the protein level to guide genomic and transcriptomic mining analyses to gain insights into a short list of gene candidates. Proteomics provides a functional genomic analysis of a biological system to guide the selection of acting enzymes. This strategy has been recently employed in the discovery of specific steps and reactions in metabolic pathways. Camptothecin (CPT), an antitumor compound, is a quinolone alkaloid that derives from the Chinese tree *Camptotheca acuminata*. Although many steps of the pathway have been previously elucidated, some tailored enzymatic steps convert the critical precursors strictosidinic acid and strictosamide into camptothecin. Many of these steps belong to CYP450-mediated oxidation enzymes. In order to decipher the responsible enzyme for the epoxidation reaction, 10,274 proteins were identified by a shotgun analysis in different *C. acuminata* tissues, including flower, fruit, leaf, seedling and stem [167]. The tissue comparison showed that the main location for synthesis was the stem. This was an unexpected result because CPT accumulates mainly in fruit. In a previous study, 12 target CYP450 genes were mined from the CYP450 candidates in plantlets by taking a targeted metabolomic and transcriptomic combined approach [87]. The proteomic comparative analyses indicated that the proteins responsible for CPT biosynthesis were enriched in the stem samples. Two CYP71 proteins were identified and one clustered with the genes and proteins responsible for the downstream pathway for CPT. The proteomic study results served to prioritize CYP450 candidates from dozens of candidate genes and to guide the candidate mining from genomic and transcriptomic resources. A phylogenetic analysis showed seven genes that clustered with an epoxidase from *C. roseus*. Finally, four candidate genes with a matching expression profiled with the CPT pathway enzymes were further functionally characterized. This provided evidence for one candidate of a newly characterized epoxidase CYP71. This study also revealed that the new epoxidase was responsible for the synthesis of CPT and flavonoids in *C. acuminata*. The authors hypothesized the possibility of the neofunctionalization of an ancestral CYP71. Following the same strategy, a multi-omics database with proteomic-derived data of *C. acuminata* was utilized to perform hydroxylase-oriented mining and screening. Three CYP81 genes were identified and two of them, CYP81BQ18 and CYP81B256, catalyzed CPT hydroxylation at the C-10 position in vitro and in vivo [168]. Another example of a proteomics mining approach consisted of a multi-omics analysis of the transcriptome, metabolite and proteome time series of a mulberry cell culture. It elicited that resveratrol and oxyresveratrol (OxyR) accumulated upon elicitation with MeJA and MBCD [156]. During the search for the enzyme responsible for OxyR synthesis, which remains undisclosed to date, two hypotheses were put forward: produced from either resveratrol hydroxylation or independently produced. The main hypothesis was that OxyR synthesis involves hydroxylases, of which some correspond to CYP450, which acts before or after resveratrol is produced. The transcriptomic analysis identified a complete set of elicited phenylpropanoid- and stilbenoid-related genes. These included 22 STS genes and a group of six *p*-coumaroyl-CoA 2′-hydroxylases (C2′Hs) that were highly co-expressed with resveratrol and OxyR accumulation. The label-free analysis of the proteome identified 3158 proteins, including feruloyl CoA-6′-hydroxylase (F6′H) parallel to the STS profile and metabolite accumulation. To further investigate the potential of the F6′H gene family being highly co-expressed with STS genes, a phylogenetic analysis with other hydroxylases was conducted. It concluded that the genes and proteins annotated as F6′H could act as C2′H. These authors proposed that the production of hydroxylated *p*-coumaroyl-CoA by putative C2′H enzymes under elicited conditions could be a substrate of STSs for catalyzing OxyR production. The functional characterization of transiently transformed *N. benthamiana* plants and grapevine (*V. vinifera* L.) cell suspensions validated the role of C2′Hs as the first committed step of OxyR synthesis and provided an alternative substrate for STSs, by hydroxylating *p*-coumaroyl-CoA into 2′4′-dihydroxycinnamoyl-CoA.

#### 5.1.5. Targeted Proteomics Approach

Seeking a profounder analysis is possible by a targeted proteomics approach. One of the most widely used methods for quantification is the SRM acquisition mode in a triple quadrupole instrument. As previously discussed, the main advantages of this approach are sensitivity and selectivity, which make this methodology suitable for the quantification of low abundant proteins of interest. Specific isozymes can be monitored to provide the quantification of specific proteoforms instead of canonical proteins, which are typically identified in a discovery proteomics study. One example of the potential of this technology appears in the study reported by Zulak et al. [169]. In it, the authors specifically detected and quantified 13 known members of the terpene synthases (TS) gene family and three isoforms of 1-deoxy-D-xylulose 5-phosphate synthase (DOX) in the MeJA-induced defense response of Norway spruce bark tissue. In Norway spruce (*Picea abies*), approximately 95% of oleoresin is composed of monoterpenes and diterpenoids. They are formed de novo upon either insect attack or treatment with defense hormone MeJA. The TSs of conifers are encoded by large gene families. The changes in only a few amino acids can lead to drastic changes in the terpenoid profile of a given TS enzyme. By doing so, the specific biochemical functions of individual TS family members cannot be predicted based on sequence similarity alone. Other techniques have also failed to determine the protein and transcript abundance of individual TSs using LC-MS/MS, Western blotting or hybridization-based techniques, such as Northern blotting, due to the high sequence similarity of these isoforms. Multilevel investigation by SRM in combination with qRT-PCR and GC-MS has provided more in-depth knowledge of the complex induced response of terpenoid oleoresin biosynthesis in Norway spruce. Another study has demonstrated that SRM can be sensitive and selective, but also have a high throughput technique. The authors accurately measured the levels of 221 targeted proteins contained in the glandular cell sample recovered from 100 glandular trichomes (GTs) in tomato [170]. Comparative quantitative proteomics using SRM assays of type VI trichome gland cells between different organs (leaves, green fruit, calyx) has revealed specific organ-enriched proteins.

Targeted approaches can also be employed to validate the high-throughput proteomic results as an alternative method to specific immunoassays to confirm protein accumulated levels. Thus, a PRM acquisition mode has been able to verify the levels of 12 proteins in tobacco, detected to be deregulated by iTRAQ in the same study. Of them all, seven proteins showed consistent trends with the iTRAQ quantitative results [171].

### 5.2. Studying the Interphase of Primary and Specialized (Secondary) Metabolism

Published studies into the interconnection between primary and specialized metabolism are not abundant, and most studies focus on the secondary metabolism pathways involved in a SPM of interest. Bearing in mind that the precursors for SPM biosynthesis are primary metabolites, the integral study of metabolism can provide insights into the increasing productivity of those specialized metabolic pathways. Balcke et al. (2017) [172] studied the connection between the primary and secondary metabolisms that operate in the type IV GTs in tomato. GTs can be considered highly productive cell factories that can produce and accumulate large amounts of SPMs. The authors applied a multi-omics approach, complemented with 13C-labeling experiments, to make comparisons at the metabolic, transcript and protein levels of the type IV GTs and leaves of a cultivated (*Solanum lycopersicum* LA4024) and a wild (*S. habrochaites* LA1777) tomato species. In LA4024, the major produced metabolites were monoterpenes and conjugated flavonoids, mostly rutin. When quantified, rutin contributed 25% of the trichome dry weight in LA4024. LA1777 produced mostly sesquiterpene, acyl-sugars and sesquiterpene carboxylic acids. The sum of two major sesquiterpene carboxylic acids [(+)-(E)-a-santalene-12-oic acid and (+)-(E)-endo-bergamotene-12-oic acid] came to 23% of the GTs’ dry weight in LA1777. Then, GTs were assumed to present remarkably high energy and carbon demands. To answer the above question, the integrative multi-omics analysis provided a large dataset, which allowed the authors to decipher a putative model for tomato trichome metabolic efficiency. The proteomics analysis was based on a comparative shotgun experiment that complemented the transcriptome dataset. The authors reported a slightly positive correlation between the transcriptome and proteome, with R values of 0.341 (LA1777 leaf), 0.367 (LA1777 trichome), 0.418 (LA4024 leaf) and 0.414 (LA4024 trichome). However, the enrichment analysis provided similar results in both the large transcriptome and proteome datasets. The study revealed how primary metabolism is organized to supply carbon and energy for terpenoids biosynthesis. In this model, sucrose is imported from leaves, this being the major carbon source in trichomes, and the light-dependent reactions of photosynthesis supplies energy and reducing power. Other primary metabolic pathways that implicate MEP and MVA pathways have been studied, and the increased expression and up-regulation of specific enzymes have indicated a putative key role in diverting carbon into terpenoids synthesis in GTs.

Other studies have applied proteomics to investigate how primary metabolic pathways organize and adapt in different tissues to deliver a specific SPM profile. Hassan et al. (2024) [173] studied both the proteome and metabolome profiles of oil palm (*Elaeis guineensis Jacq.*) fruit. The metabolomic analysis identified how different stilbenoids accumulated in four differential fruit tissues: pulp (mesocarp), skin (exocarp) and seed (kernel and shell). *t*-R was detected in the shell and mesocarp, while piceatannol, a hydroxylated derivate of *t*-R, accumulated in the shell, exocarp and kernel and *trans*-piceid, the glycosylated counterpart of *t*-R, did so in the exocarp. A comparative proteomics analysis allowed them to identify the enzymes involved in the biosynthesis of stilbenoids, including *t*-R di-*O*-methyltransferase (ROMT), which was highly expressed only in the exocarp, mesocarp and shell tissues. Other enzymes that act upstream of stilbenoid biosynthesis, such as those located in the phenylpropanoid pathway, and other primary metabolic pathways, such as glycolysis and the tricarboxylic (TCA) cycle, were up-regulated in mesocarp and exocarp tissues. This study provided information about tissue-specific cellular functions for primary and secondary metabolisms in agreement with metabolome profiles.

Specialized (secondary) biosynthetic pathways are linked with the primary metabolism, which provides compound derivatives that serve as substrates for PTM biosynthesis. The comprehensive study of the metabolic pathways to link downstream SPM biosynthesis with the primary metabolism, including central and energy metabolisms, provides an understanding of how plants organize the core metabolic network to support SPM production. This provides valuable knowledge that is applicable in metabolic engineering to produce pharmaceutical, agronomical and bioactive SPM compounds of interest.

### 5.3. Studying the Regulation of SPM Metabolism

In order to understand SPM production well, it is crucial to unravel the regulatory mechanism that controls their biosynthetic pathways and the external signals that affect them. However, the exploration of regulatory networks is still limited by the amount of complete biochemical pathways of the specific metabolites known to date [174]. The pathway regulation of SPM metabolism is affected by the control of gene expression at their multiple regulation levels (transcriptional and posttranscriptional processing, translational and posttranslational processing), but also by the multiple interactions that occur due to protein assemblies (protein–protein interactions) or the effect of a metabolite on regulatory enzymes (protein–metabolite interactions). In this section, we focus on the contribution of proteomics to the SPM regulation that involves analyzing PTMs; that is, the posttranslational modification that affects the activity of many enzymes, and the identification of other regulating proteins of interest as the transcriptional factors involved in the transcriptional regulation of SPM metabolic processes. Recently, reviews in the literature have covered other topics of the regulation of SPM metabolic pathways regarding their regulation during the transcriptional process [174] or to study interactomes [175,176]. The regulation of metabolic pathways is a complex process that involves multiple genes, protein species and posttranslational modifications. Proteomics, mainly through the MS-based determination of proteins, is a tool capable of investigating chemical modifications, PTMs, that originate from the same gene, different proteins or proteoforms [72], and each one may affect their activity or function. Research into PTMs has significantly contributed to understanding how plants respond to and tolerate stress [129,134,135,137,177]. However, publications about the study of PTMs in the SPM metabolism context are still scarce.

Phosphorylation is critical in mediating signal transduction pathways by modulating protein activity, stability and conformation, subcellular localization and the protein–protein interaction (PPI). Although protein phosphorylation is the most widely investigated PTM in plant biology research [123,124,129], some examples appear in the bibliography that focus on SPM metabolism. Wang et al. (2020) [178] studied the regulatory mechanism that lies behind cucumber (*Cucumis sativus L.*) resistance to parasitic nematode infection by the root-knot nematode (RKN) [Meloidogyne incognita Kofoid and White (Chitwood)]. The authors applied an integrated high-throughput strategy that included transcriptomic, proteomic and phosphoproteomic approaches to gain insight into plants’ molecular responses against biotic stress. They investigated changes in the transcription, protein and protein phosphorylation levels between resistance line IL10–1 and susceptible line CC3 at 3 days postinoculation (3 dpi) with *M. incognita.* The quantified genes and protein species showed low consistency in both IL10–1 and CC3, with a value of R = −0.0385 and 0.0796, respectively. A small fraction of deregulated proteins was detected in the proteomic experiment. Between 47% and 51% of the deregulated proteins displayed a similar trend with their corresponding transcripts, whereas the other members showed opposite trends. Data indicated that gene expression cannot fully represent the abundance of protein species and pointed out the existence of the posttranslational regulation of protein translation. The proteomic experiment consisted of the relative quantification of the proteome using the isobaric chemical tags of proteins by iTRAQ. Then the labeled phosphopeptides were enriched based on TiO_2_, which led to the identification of 1870 phosphoproteins containing 3302 phosphorylation sites. The enrichment analysis of the proteome showed that the flavonoid pathway was significantly enriched in IL10–1, which indicates its important role in IL10–1 against *M. incognita*. The analysis of the deregulated phosphoproteins pointed out the involvement of the MAPK signaling pathway upon nematode infection, which potentially triggered flavonoid production linked with the cucumber resistant phenotype. Two other phosphoproteomic studies revealed the activation of the MAPK signal transduction pathway by abiotic stress, which led to SPM production in the plant. Liu et al. (2022) [179] studied the effect of UV-B radiation on the regulation of pathways, which led to SPM accumulation in *Mahonia bealei* leaves, mainly flavonoids and alkaloids in stems and roots. They analyzed the enriched peptides in TiO_2_ by label-free quantitative proteomics and identified 92 phosphorylation sites in 148 proteins. The quantified phosphoproteins up-regulated by UV-B radiation were related to plant hormone brassinosteroid signal transduction and the MAPK signaling pathway, and to the primary metabolic pathways of photosynthesis, glycolysis, the TCA cycle, carbon fixation, and the synthesis and metabolism of amino acids, including Tyr and Phe, precursors of flavonoids and alkaloids. These results suggest that the combined action mechanism of phosphoproteins in the photosynthetic-glycolysis-TCA cycle pathway and the amino acid metabolism may consequently provide the substrate and energy for the synthesis of secondary metabolites. Pi et al. (2018) [180] quantified 4697 phosphorylated sites from 2239 phosphoproteins by applying a frequent proteomic workflow to analyze phosphoproteins based on peptide labeling by iTRAQ, phosphopeptide enrichment by TiO_2_ and an LC-MS analysis. Of the 412 quantified phosphopeptides, TF GmMYB173 was differentially affected by NaCl. GmMYB173 regulates flavonoid syntheses by the expressional control of GmCHS5, a chalcone synthase. Those authors confirmed the direct involvement of this TF by comparing the phosphoproteomes of the created transgenic roots for GmMYB173 and GmCHS5 genes, and the metabolic profiles of dihydroxy B-ring flavonoids. Hence, through the regulation of flavonoid syntheses, CHS acts as a hub in the complicated network, which leads to the establishment of soybean tolerance to salinity.

Lysine succinylation, a widespread modification found in various organisms, plays a critical role in regulating secondary metabolism in plants [136]. You et al. (2023) [181] performed a proteomic analysis to identify lysine succinylation sites using affinity purification followed by HPLC-MS/MS in the medicinal plant *Salvia miltiorrhiza* Bunge. By this strategy, 53 types of enzymes were identified as succinylated proteins, including PAL and aldehyde dehydrogenase (ALDH). They observed that PAL, a crucial enzyme involved in the biosynthesis of rosmarinic acid and flavonoids, displayed two succinylation sites.

Lysine acetylation is the third most common form of PTM after phosphorylation and ubiquitination and is well-known for its involvement in protein–protein interactions by affecting protein interaction with DNA, enzyme activity and protein localization [182]. Changes in cellular lysine acetylation have been reported by Li et al. (2021) [183], which could regulate various aspects of primary carbon and nitrogen metabolism, as well as secondary metabolism in paper mulberry. A lysine acetylation proteomics analysis of paper mulberry seedling leaves has been conducted in combination with iTRAQ labeling, which was accurately identified by LC-MS/MS after the fractionation of peptides by high pH reverse chromatography.

TFs are key regulators of both intrinsic and extrinsic signals. Their detection in proteomic analyses is challenging because they tend to be low-abundant at the protein level. Another characteristic of TFs is that their function depends on PTMs, frequently by phosphorylation, and also on their interaction with other proteins [184]. TFs have been largely underdetermined in most proteomic studies. However, MS-based advances in the last few years, together with the development of sensitive methodologies, have allowed us to quantify the TFs implicated in regulating biological processes [185]. Various transcription factors, such as DREBs, MYCs, WRKY and MED, which uniquely influence terpenoid biosynthesis, have been identified through the differential expression by Q-TOF LC-MS/MS upon jasmonate acid (JA) elicitation of *Andrographis paniculata* plantlets [92]. Proteins from the MAD-box family accumulate in the pequi proteome (*Caryocar brasiliense* Camb.), a fruit characterized by the high content of carotenoids and other SPMs, such as trigonelline alkaloids. MADS-box protein GGM13 and MADS-box TF 25 show increased accumulation by 35.5- and 5.5-fold of the ripening phase of development, respectively [82]. Finally, 46 TFs have been identified in a comprehensive proteomic approach of a developing rice stem internode. The C3H family is the most common, and CDC5 (LOC_Os04g28090) from the R3-MYB family and NAC2 (LOC_Os08g06140) from the NAC family are implicated in cell wall-related or cell division-related TF families [186].

### 5.4. Studying the Effect of Biotic and Abiotic Stress on SPM Biosynthesis

Plants are affected by biotic and abiotic stress due to their sessile nature. Therefore, different stresses, such as salinity, heavy metals, water (drought and flood), temperature (high or low) and UV-B radiation, and environmental factors, such as high CO_2,_ ozone or pathogenic attack, may impact their growth and development. A stress response initiates when a plant recognizes stress at the cellular level, which activates the corresponding signal transduction pathways to change certain genes transcription in a plant integrated systemic response. That induced response to stress modifies growth, development and reproductive capabilities in addition to SPM biosynthesis. Plants’ ability to withstand stress is connected to their increased synthesis of most SPMs as part of their chemical defense response mechanism [187]. So, SPMs are crucial for plant–environment interaction and are part of the plant’s response to adapt to a changing environment [3].

Plant responses to stress, of either an abiotic or biotic origin, have been extensively studied via proteomics [123,188,189]. The role of proteins in the plant response to stress is critical, because they directly participate in the adaptation of plant physiological characteristics by setting a novel phenotype. Furthermore, we ought not to forget that proteins are the main executors of cellular processes, and their function is critical for the maintenance of cellular homeostasis. So, it is not surprising that many studies have applied proteomics in plant stress research. The main approach followed when studying effects of stress on plants has been comparative proteomics by label-free [190,191,192,193,194,195,196,197,198,199,200,201] or label-based quantification using isobaric tags by iTRAQ [195,202,203,204,205,206,207,208,209,210,211,212,213,214,215,216,217,218,219,220,221,222,223], TMT [224,225,226] or iBT [227]. Another shotgun analysis has been applied by advanced MS acquisition via DIA [228,229,230] or SWATH [106,231] MS. Gel-based comparative proteomics by 2-DE [232,233,234,235,236] has also proved useful. In all the found publications, a set of deregulated proteins belonging to the plant secondary metabolism has been detected, or the term ‘secondary metabolism’ has been enriched during their functional analysis. Here, we highlight examples of research conducted using proteomics to reveal changes in those proteins involved in plant-specialized metabolism induced by stress.

#### 5.4.1. Abiotic Stress

One of the main abiotic factors to affect plant growth and development is drought stress (DS). Studies into plants during droughts, which often lead to oxidative stress, have higher levels of SPMs. In grapevine roots, the comparison of two genotypes, one of low (101.14) and another of high tolerance (M4), has shown an increase in the enzymes involved in the synthesis of flavonoid (CHS, 5GYT) and stilbene (STS) compounds in M4 [190]. They concluded that the capability to synthesize larger amounts of antioxidant compounds, such as flavonoids and stilbenes (i.e., resveratrol), enhanced tolerance to drought in M4. In another study, a panel of drought protein markers was selected by running a hypothesis-free study for *Quercus ilex*. seedlings. The enzyme CHS was one of the selected markers, whose protein profile was consistent with the increased phenolic levels that the authors observed in a previous research work [192]. In another study about the same forest tree, the enzymes involved in secondary metabolism (PAL, flavanone 3-hydroxylase (F3H), CCoAOMT) were identified as being drought-responsive in roots, and consistently with the increase in free phenolic content in roots [232]. When studying DS in faba bean leaves, DFR was up-regulated; DFR is an enzyme involved in the biosynthesis of flavonoids [198]. Qiu et al. (2021) found out that fulvic acid enhances resistance in tea plants to DS by increasing phenylpropanoids (C4H, 4CL) and triterpenoids biosynthesis [202].

Salt stress (SS) produces an enhancement of the key enzymes of phenylpropanoids (C4H) and flavonoid (F3H) pathways in *Beta vulgaris* [203]. In another research work, Jia et al. (2019) showed that the most important factor in the leaves of the mycorrhizal *E. angustifolia* seedlings was the increased abundance of secondary metabolism-related proteins, especially the metabolism of the proteins involved in phenylpropanoids (HCT, CAD, CCoAOMT) and flavonoids. The comparison of salt-tolerant (ZM1) and salt-sensitive (A17) Medicago species under salt stress also revealed an increase in phenylpropanoid enzymes (CAD, CCoAOMT) and flavonoids, specifically isoflavonoids (IFS) in ZM1, which was better able to remove reactive oxygen species (ROS) presumably by the enhancement of the antioxidant system and secondary metabolism [217]. Some halophilic species thrive on salinity levels that are toxic to most plant species. To study the molecular mechanisms of the halophilic tree mangrove *Kandelia candel* to cope with salinity levels, Wang et al. (2016) conducted a multilevel analysis via proteomics, transcriptomic, targeted metabolite and gene PCR-RT analyses. The large proteomics and RNA-seq dataset revealed the up-regulation of the following: the first three enzymes of the core phenylpropanoid pathway (PAL, C4H and 4CL); flavonoid biosynthetic enzymes (CHS, F3H, flavonoid 3′-monooxygenase (F3′H), flavonol synthase (FLS)); anthocyanin and leucoanthocyanin biosynthetic enzymes (ANS, ANR, LAR). They demonstrated a parallel increase in the accumulation of phenylpropanoids and their derivatives, phenolics, flavonoids, anthocyanins, proanthocyanins and lignins in salt-stressed *K. candel.* These authors emphasized the importance of these secondary compounds in not only this tree halophyte’s tolerance to a mild salt environment, but also in its adaptation to high salt environments [221].

Plant cells have mechanisms in place that avoid the hazardous accumulation of certain metals, especially toxic metals, because if these metals appear in excess, they are detrimental to plants. SPM generation under heavy metal stress has been the subject of recent research works [237]. At physiological and metabolic levels, heavy metals impact the synthesis of various photosynthetic pigments, carbohydrates, proteins and non-protein thiols in plants. *Panax ginseng* and *Panax quinquefolius* can survive for long periods in iron toxicity-stressed environments. A proteomic comparison in both species under Fe (Fe(II)-EDTA) toxicity showed the up-regulation of many proteins associated with redox reactions and the pathways involved in ginsenoside synthesis (3-hydroxy-3-methylglutaryl-CoA reductase (HMGR), UDP-glycosyltransferases (UGT and CYP450), phenylpropanoid and lignin synthesis (PAL, HCT, CCoAOMT, CCR, C4H and CAD), flavonoid synthesis (LAR), isoflavone synthesis (2-hydroxyisoflavanone dehydratase (HIDH), isoflavone-7-*O*-methyltransferase (7-IOMT) and isoflavone 2′-hydroxylase (CYP81E1_7) [214]. In another research work, aluminum- (Al) induced root proteomic changes in switchgrass were related to the increase in the enzymes involved in lignin synthesis [226]. In Poplar, the up-regulation of the enzymes from lignin (CAD, COMT) and flavonoid biosynthesis (PAL, 4CL, CHS, F3H) pathways indicated that these pathways were strongly activated upon lead (Pb) exposure [181]. *Sedum plumbizincicola* is a cadmium (Cd) hyperaccumulating herbaceous plant that can accumulate large amounts of Cd without being affected. The transcriptome and proteome analysis found that the pathways involved in oxidative phosphorylation, phagosome, glutathione metabolism and phenylpropanoid biosynthesis were significantly enriched biological functional pathways. A protein, annotated as flavonoid 3′,5′-methyltransferase (FAOMT), was strongly up-regulated. Functional characterization via a transgenic line overexpressing FAOMT demonstrated its involvement in lignin biosynthesis [225]. Supported by previous studies, these authors indicated that one of the primary components of plant cell walls, lignin, plays a crucial role in resistance to heavy metal stress. Excess heavy metal is detoxified by its chelation by cell walls, or phenylpropanoid may also have chelation activity. Plant responses to increased arsenic (As) concentrations have also been studied via proteomics. Two *Brassica napus* cultivars, ZS758 and Zheda 62, with a different tolerance and response to As, showed the deregulation of the proteins participating in secondary metabolism, enzymatic antioxidants, defense and redox homeostasis. An increased abundance of enzymes participating in secondary metabolism, such as PAL and CHI, was observed. The authors concluded greater tolerance in ZS758 depending on a multilevel coordination of a more efficient energy metabolism and defense upon proteomic and physiological results [218]. The same *B. napus* cultivars treated with MeJA before being exposed to As showed that CAD, CHI and IFR abundance rose in cultivar S758, while only CAD was up-regulated in cultivar Zheda 62. The deregulated proteins involved in stress defense and photosynthesis were up-regulated in both cultivars, together with the activation of secondary biosynthesis, which indicates an important role of MeJA in mitigating adverse As stress effects on *B. napus* [210].

The plant defense system is immediately triggered by UV-B irradiation, particularly the production of the metabolites and enzymes involved in the UV-B response. Studies carried out under UV-B stress report increased SPM production. In *M. Alba,* which has been studied in a UV-B-dark regime, the abundance of five SPMs significantly increased, namely moracin N, moracin C, morachalcone, chalcomoracin and guangsangon E [105]. The authors indicated that UV-B radiation can be a potential approach to improve SPM contents in plants, notably for *M. alba*. In *M. bealei* leaves, a combined UV-B and darkness effect was analyzed by combining proteomics and metabolomics. Significantly changed metabolites were overrepresented in phenylalanine metabolism, phenylpropanoid, flavonoid and alkaloid biosynthesis. Some new alkaloids, such as indole alkaloids, acridone alkaloids, piperidine alkaloids, purine alkaloids and tropane alkaloids, were identified for the first time in *M. bealei* leaves, besides isoquinoline alkaloids, which were induced by UV light exposure. A protein–protein interaction network revealed a close connection among the TCA cycle, glycolysis, nitrogen metabolism and secondary metabolism. After the validation of protein abundances via PRMs, the proteomic analysis indicated an enhanced TCA cycle to provide intermediaries for the biosynthesis of N compounds and secondary metabolites [199]. Gong et al. (2023) applied an integrative metabolomic and proteomic strategy in *Rhododendron chrysanthum* with UV-B and ABA applications. Phe considerably increased primary metabolism, which indicates the role of Phe to support the carbon shift to increase SPM production. Particularly, carbon compounds C6C1 (phenolic acids) and C6C3C6 (flavonoids and flavonols) were up-regulated at the expense of carbon compounds C6C3 (cinnamic acid and ferulic acid). The key enzymes of Phe, Tyr and Trp, the flavonol metabolism pathway, the flavonoid metabolism pathway and the monolignol biosynthesis pathway were deregulated. The functional analysis based on an enrichment analysis, and the correlation between deregulated proteins and metabolites, revealed that enzymes CHS, CHI and F3H and chorismate mutase affected the levels of primary and secondary metabolites [207]. The authors hypothesized that external ABA application could work in concert with UV-B to facilitate the transformation of primary metabolites into phenolic compounds. In another integrated metabolic and proteomic study, the UV-A treatment of *Taxus chinensis* var. mairei leaves indicated increased paclitaxel production in the coordinated accumulation of the enzymes of glycolysis and secondary metabolism. These results suggest the activation of glycolysis, which leads to an increase in glyceraldehyde-3-phosphate and pyruvate, which are substrates in the first MEP pathway step. 1-Deoxy-d-xylulose-5-phosphate reductoisomerase (DXR) and 4-hydroxy-3-methylbut-2-enyl diphosphate reductase (HDR) increased the key enzymes of the MEP pathway, which provided precursors for paclitaxel, phytosterol and carotenoid biosynthesis [235].

Tropospheric ozone (O_3_) pollution is another significant factor influencing plant metabolic responses because this gas can be phytotoxic to plants given its properties as a strong oxidant [3]. The proteomic profiles of two soybean genotypes with different O_3_ resiliences, Fiskeby III (O_3_-tolerant) and Fiskeby 840-7-3 (O_3_-sensitive), revealed under O_3_ stress an increase in the enzymes participating in the synthesis of polyamines, polyamine alkaloids and alkaloids in Fiskeby 840-7-3, while lignin biosynthesis was activated in Fiskeby III [196].

Cold stress (CS) by either chilling (0–15 °C) or freezing (below 0 °C) negatively affects plant growth and development. An integrated transcriptomic and proteomic approach detected similar trends in both analyses with PAL and ANS rising in loquat fruit (*Eriobotrya japonica* Lindl) at chilling temperatures. This scenario suggests the synthesis of anthocyanins [216]. In another study with two rapeseed cultivars, another integrated transcriptomic and proteomic approach with freezing-resistant (NS) and sensitive (NF), demonstrated the enhancement of the enzymes involved in phenylpropanoid biosynthesis in NS under freezing stress [229]. With *Citrus junos* under freezing stress, Jiang et al. (2020) reported an increase in phenol content, but a drop in flavone content, and the total flavonoids content did not change upon CS. At the protein level, there was an increase in phenylpropanoids (HCT, PAL) and the flavonoids pathway (CHS, CHI, IFS, IFR) [224]. Conversely, heat stress promoted isoprenoid metabolism in spinach [209] and a high turnover rate for the enzymes involved in secondary metabolism in *A. thaliana* shoots, which were analyzed after metabolic labeling by ^15^N-stable isotopic protein labeling [238].

Inorganic phosphate (Pi) deficiency significantly limits plant growth in natural and agricultural systems. A common response of *A. thaliana* to Pi deficiency is the accumulation of anthocyanins in shoots. The proteomic comparison showed a deregulation of ANS, DFR, PAL and anthocyanidin 5-O-glucosyltransferase (AA5GT), all of which were enriched in the GO term on the ‘flavonoid pathway’. DFR catalyzes the reduction of dihydroflavonols to leucoanthocyanins (Figure 1), a rate-limiting step in anthocyanin and proanthocyanidin biosynthesis. So, the authors further characterized the potential role of the flavonoid pathway by analyzing a DFR null mutant (*tt3*) defective in anthocyanin biosynthesis. The *tt3* mutant under Pi deficiency showed an increased expression for the genes acting upstream of DFR (PAL, 4CL), but a lower expression of downstream genes LDOX and AA5GT. The authors concluded that anthocyanin accumulation depends on DFR, but Pi deficiency could initiate the flavonoid biosynthetic pathway [213]. Selenium (Se) is a prosthetic group of many enzymes and plays an important role in alleviating oxidative stress and immunity. Se uptake in a diet is fundamental, and Se fertilization is used to enhance Se in the human diet. To study the Se response in wheat, Feng and Ma (2021) studied protein and transcript changes in seedlings. Of the 11,656 identified proteins, 273 were deregulated upon Se treatment. Secondary metabolism, ROS scavenging and carbohydrate metabolism were significantly enhanced, and 14 UFGTs, those responsible for the last anthocyanidin biosynthesis step, were up-regulated at the protein level [204].

In nature, however, simultaneous or sequential exposure to stress is a more realistic scenario to which plants must adapt, and the study of combinations of stress is reported in the literature [3,189]. The effects of high CO_2_ and temperature have been analyzed in *Picrorhiza kurroa*, a medicinal plant endangered by climate change. The total protein, phenolics, flavonoids and antioxidant activity were altered in a tissue-specific manner to high CO_2_ and temperature. The proteomic experiment detected the up-regulation of CYP 86B1, UGT73B3, S-adenosyl-L-methionine:trans-caffeoyl-coenzyme A 3-O-methyltransferase (SAM:COMT), which are involved directly or indirectly in picroside biosynthesis, together with a marked rise in picroside, a type of iridoid glycosides [234]. In another study, the combined effect of high CO_2_ and Fe deficiency showed an increase in the flavonoid pathway in soybean via proteomics [197].

The effect of high altitude can be considered a combined environmental factor constituted by distinct pressure stresses on plants because they are exposed to different temperatures, UV exposure, oxygen levels or atmospheric pressure compared to non-high altitudes. Li et al. (2024) reported that the photosynthetic and biosynthetic enzymes (PAL, 4CL, CHS) and NADPH-dependent carbon double bond reductase (CDBR) related to the production of phloretin, a dihydrochalcone (DHC), were down-regulated and up-regulated, respectively, which led to DHC accumulation and lower chlorophyll content in *Malus hupehensis* Rehd leaves. [230]. The proteomic study also allowed the identification of a novel glucosidase (*Mh*GH2) that accumulated considerably at high altitude. *Mh*GH2 presented greater catalytic efficiency and higher binding affinity toward the glycosylated form of phloretin, phloridzin, compared to the already known glucosidases in other species. These authors highlight that their results provide new knowledge about phloretin accumulation in plants.

Wounding stress (WS) can occur at post-harvesting during storage or transportation. The plant response to WS is a complex process that generates physiological modifications to protect wounded tissue. The proteome of wounded broccoli (*Brassica oleracea* L. var. italica) at different intensities shows the activation of phenylpropanoid biosynthesis (PAL, 4CL, CAD, COMT, CYP73A) and glucosinolate biosynthesis. The up-regulation of carbohydrate metabolism, starch and sucrose metabolism, and the amino acid metabolic pathways of phenylalanine metabolism, cysteine and methionine metabolism, branched- amino acids degradation, may all provide the precursor for SPM biosynthesis [239].

#### 5.4.2. Biotic Stress

Living beings, including arachnids, bacteria, fungus, herbivores, insects, nematodes, oomycetes, viruses and weeds, may cause biotic stress. SPMs are produced by plants in response to biotic stress [240]. A wild eggplant *Solanum sisymbriifoliumin,* known to be resistant to *Verticillium dahlia*, a fungal pathogen that often infects eggplant (*Solanum melongena* L.), has been studied at the protein level via proteomics to understand the mechanisms for its resistance [222]. After 12 and 24 h, the infected roots showed a deregulation of the proteins participating in the phenylpropanoid pathway, cell wall organization and reinforcement-related proteins, phytohormones-signal-pathways-related proteins, and other defense-related proteins [222]. In another study, in the needle proteome of a susceptible (*Pinus radiata*) and a relatively resistant (*Pinus pinea*) species upon *Fusarium circinatum*, the causing pine pitch canker (PPC), inoculation was analyzed by GeLC-MS/MS [241]. In susceptible *P. radiate,* secondary metabolism activation was induced of the shikimate pathway and the phenylpropanoids pathway and lignans (CAD, (+)-pinoresinol reductase) biosynthesis. In resistant *P. pinea*, antioxidant SPM like flavonoids seemed enhanced (CHI). Teng et al. (2021) studied the mechanisms that lie behind *Brassica rapa* ssp. Pekinensis resistance to *Sclerotinia sclerotiorum* after melatonin (MT) treatment. An integrated metabolomic and proteomic analysis of the MT treated in infected leaves showed that amino acid metabolism was activated together with antioxidant enzymes and the others involved in secondary metabolism, which led to glucosinolate accumulation [242]. To explore the response of soybean resistance after being exposed to the larva of *Lamprosema indicate*, a major leaf feeding insect, Zheng et al. (2017) carried out a proteomic analysis of high resistance and susceptible varieties against *L. indicate* [223]. Deregulated proteins were related to SPM biosynthesis as flavonoids, phenylpropanoids and flavanol and flavone biosynthesis.

Plants employ a complex molecular network that regulates cellular processes to adapt to a changing environment. Hormones and transcriptional regulatory events are fundamental components of that molecular network and regulate specialized metabolic responses. Indeed, the effect of the hormones that modulate plant responses to stress have been studied using proteomics approaches [207,210,233]. The identification of the TFs induced by stress by proteomics has also been reported. Fifteen TFs were detected to be up-regulated by heat stress in spinach leaves [209]. TFs, GATA-TF and ABI5/ABFs were found to be up-regulated in *Vicia faba* leaves in response to drought stress [198]. Several Al-responsive TFs, such as basic-leucine zipper (bZIP), the TF family protein, nuclear factor Y (NFY) and the C2H2-like zinc finger protein, were identified in Switchgrass roots [226]. The PTM analysis of proteins in response to stress is also relevant [189].

All the above scenarios suggest the benefits of abiotic and biotic stress on the biosynthesis of certain SPMs, and may provide an approach to improve overall or targeted SPM production via the manipulation of environmental parameters, such as temperature, water availability or UV-B exposure. In fact, using biotic stressors as elicitors is a very efficient strategy that is adopted to either increase SPM production or to study plant-specialized metabolism, as discussed in the above section [15,240]. Another approach that could support the study of certain specialized metabolisms is the comparison of different genotypes (i.e., resistant vs. susceptible) to reveal their adaptation to the environment.

## 6. Challenges and Future Perspectives

Most SPMs are not widely distributed in the plant kingdom. Instead, they accumulate in taxonomically restricted species. Only a few model species can be employed to study SPMs with advanced genetic features, such as easy transformability and the availability of mutant collections [1]. Some examples are *Arabidopsis*, which possesses many conserved major secondary metabolic pathways like phenylpropanoids and triterpene, *M. periwinkle* for alkaloid biosynthesis studies, or *Medicago* for isoflavonoid and triterpene biosynthesis studies. However, they lack the enzymatic steps involved in the synthesis of these classes of compounds for other species. Therefore, SPM biosynthetic pathways of interest occur in non-model plant species, which are also underrepresented in the available databases. The study of SPM metabolism via proteomics has been hampered in the past by the lack of sufficient sequence data in the search space, and this may still act as a limiting factor for some species. However, the study of SPM biosynthesis through omics-approaches in non-model species has increased considerably in the last 10 years. The increase in genomic and transcriptomic resources allows more and more non-model species to be used to study specific SPM biosynthesis [243]. It has been reported that 798 genome assemblies are publicly available in land plant species [244]. Yet, when genome sequences are lacking or incomplete for a certain organism, RNA sequencing can generate a predicted protein sequence specific-species database. Indeed omics-driven research through RNA-sequencing transcriptomics has provided a parallel approach to support protein identification by MS in proteomics studies [155,245,246,247]. Customized protein databases can be generated using RNA-seq to provide a specific database for a specific sample by increasing the number of identified proteins, but can even provide a single-cell resolution proteome database given current available technology [110]. Recently, a massive transcriptome sequencing of 1342 samples has become available, which represent 1173 green plant and other chloroplastic species that cover all the major taxa in Viridiplantae [248]. Thus, the genome and RNA sequencing of species do not seem to be a major limitation to proteomics, but rather to sequence annotation. Accurate annotation is critical to study plant-specific SPM pathways via proteomics. When orthologs in well-annotated species do not exist, careful interpretation and manual curation are needed before interpreting the proteomic data from homology-based assignments The transcriptome data can be employed to improve genome annotation quality [5,156]. Proteomics can also assist the genome annotation task by means of a proteogenomic strategy, which implies the experimental validation of a genome assembly [243]. For example, in *Cannabis* sp., a comprehensive proteome draft has been published based on a proteogenomic approach [101]. Peptides from flowers have been identified by LC-MS/MS, and the authors developed a pipeline, which is applicable to any plant material, using both ‘next-generation’ DNA sequencing (NGS) output and shotgun proteomics data to produce annotated FASTA files, which completely does away with the need for annotated genetic information. The output of this pipeline in *Cannabis* consists of 13,850 non-redundant sequences with putative annotations. An improvement in incomplete annotated genomes has been reported in different plant species via a proteogenomic approach using RNA-seq combined with shotgun proteomics data to provide a spatial resolved proteome database of 15 major sweet cherry (*Prunus avium* L., cv ‘Tragana Edessis’) tissues [102] and 24 pear tissues (*Pyrus bretschneideri*) [103]. A similar strategy has been followed for the medicinal plant *Artemisia annua* [249]. Alternatively, the standard search pipeline, based on publicly available protein databases by employing a homology-based search, may provide a first list of identified proteins [250]. In addition, other searching strategies may increase the number of protein identifications as open searches with wide mass tolerances [251] by combining the database search with the interpretation of unmatched spectra by de novo sequencing or using only de novo sequencing when MS spectra searches are not possible [252].

Another challenge that proteomics can face when studying plant-specialized metabolism is a lack of correlation between protein and metabolite profiles, as seen in the transcript, which may be in part related to the regulation of the activity of enzymes or the complex compartmentation of metabolism between parts of the cell or between cells [253]. Then, the study of plant-specialized metabolism under an omics-driven approach can be complemented by fluxomic analysis. Fluxomics focuses on quantifying the flux of metabolites through metabolic pathways and integrates this information with other omics datasets (proteomics, transcriptomic and genomics) to probe the corresponding gene-RNA-protein-metabolite interaction networks in actual time [254].

With a suitable proteomics workflow, MS can measure endogenous peptides, including bioactive PNPs, via a peptidomics analysis. Peptidomics is an emerging field that employs modern proteomics for the analytical determination of the abundance and identification of endogenously produced peptides [255]. Here, sample preparation and the experimental workflow differ from the classic proteomics analysis. Extracted peptides need further concentration and enrichment using SPE, molecular weight cut-off filtration devices or other clean-up techniques. The chemical derivatization and linearization of peptides may also be required for the case to study cyclic cysteine-rich peptides [57,256]. Peptide separation by different chromatographic separation technologies can be coupled to MS. So current MS capabilities are applied to carry out a peptidomic analysis, including TOF, quadrupole, ion-trap and orbitrap. Shotgun peptidomics, by either DDA or IDA, is applied for peptide identification purposes. As PNPs are an RiPP class of peptides, a proteomic search pipeline is generally applied for mass spectra interpretations. Few specific peptides databases are available for plants [257]. Nonetheless, other MS technologies not widely applied to plant proteomics have been reported as valuable tools for peptidomic analysis. For example, the top-down de novo sequencing of peptides is the best choice for sequence determination in some cases, including for highly modified peptides. In this sense, Fourier transform ion cyclotron resonance mass spectrometers (FTICR-MS) and high-resolution MS instruments have been proposed as suitable powerful instruments for peptide mapping via de novo sequencing [258]. A successful determination of endogenous peptides from poplar tissues in interaction with the ectomycorrhizal basidiomycete *Laccaria bicolor* allowed the identification of 1,660 peptides, applying a novel peptidomic approach [259]. Another technology generally used for metabolomic analysis is matrix-assisted laser desorption/ionization (MALDI)-MS imaging (MALDI-MSI). This technology allows for the spatial mapping of molecules. MALDI-MSI has also been successfully applied for the characterization and location of Medicago endogenous peptides [58] and cyclotides in petunia [260].

Another developing field in which MS-based proteomics technology can advance SPM metabolism knowledge is the analysis of PPIs. PPIs aim to identify protein complexes. The proteome is a highly structured entity in which proteins function in either physical or functional association with other proteins or molecules [79]. Protein interactions, together with possible multiple proteoforms, extend proteome functionality. The existence of metabolomes, enzyme-enzyme assemblies that mediate substrate channeling, in the synthesis of many SPMs, including phenylpropanoids, alkaloids, flavonoids, isoflavonoids or terpenoids, has been proposed [176]. PPI studies employ techniques like yeast two-hybrid screening, affinity-purification MS (AP-MS) or co-expression profiling to establish binary connections and complex network topologies [261]. AP-MS experiments involve a bait protein used to pull-down interactive proteins, which is followed by their MS analysis. Another hybrid approach complements MS with other structural techniques, such as X-ray crystallography and nuclear magnetic resonance (NMR) [79], which allow the identification of the subunit interfaces, topology, conformation and structure of protein complexes. AP-MS is a versatile technique where, instead of protein, the bait can be modified peptides, oligonucleotides or small molecules [79]. Also, the analysis of protein-metabolite interaction can help to understand biological regulatory processes, and it offers a potent perspective to study specific metabolite bioactivities as recently reviewed [175].

Chemical proteomics is another emerging field branch of proteomics that can offer novel techniques to study SPM metabolism. Activity-based protein profiling (ABPP) employs small molecules as probes to directly interrogate the protein function in complex proteomes. ABPP can be applied to detect specific enzymatic activities by using activity-based probes (ABPs) to react with the active sites of proteins selectively and covalently. So those enzymes can be identified by MS-based proteomic workflows [262]. This approach is successful for identifying active glycosidases during stigma development in saffron and other hydrolases upon biotic stress [263]. This technology could have an enormous impact on the identification of the missing enzymes of biosynthetic pathways and many unknown decorating enzymes that produce plenty of interesting SPMs.

The involvement of multiple cellular compartments in the biosynthesis of many SPMs is ubiquitous. This is the case of the many alkaloids by which biosynthetic pathways are generally segregated [264]. For example, morphine biosynthesis is divided into three cell types [141]. In other cases, even metabolites have to travel long distances, and alkaloids of Solanaceae are produced in roots and also accumulate in leaves. Another non alkaloid SPM biosynthesis involves different cell types and organs (e.g., cyanogenic glucosides, glucosinolates) [17]. The identity of the pathway intermediates and/or SPMs that undergo intercellular transport is not completely known for most pathways. Moreover, trafficking between different compartments implies the existence of transporters. In recent years, single-cell omics have emerged as a promising strategy to tackle the spatial–temporal coordination of SPM biosynthesis. To effectively characterize cell-specific biosynthetic pathways, the analysis of single cells instead of a whole tissue provides the required spatial resolution. Whereas single-cell transcriptomic [265,266] research has been already applied to study SPM biosynthesis, technical difficulties in single-cell proteomics profiling have precluded the same high-throughput as sc-transcriptomics. Recent advances made in single cell isolation [267] and sample preparation to analyze very low protein content samples have allowed the recent application of single-cell proteomics in plants [110,268,269,270,271]. Montes et al. (2024) have demonstrated the feasibility of sc-proteomics to differentiate the protein profiles of two adjacent root cell types as endodermis and cortex cells in *Arabidopsis* [268]. Therefore, sc-proteomics can be immediately applied to more profoundly study the SPM metabolism. Unlike mRNA levels, as discussed in previous sections, given the unique properties of the proteome that proteomics can capture, sc-proteomics will provide unique information, and combined with sc-transcriptomics and sc-metabolomics [272,273], will allow the real integration of omics-data.

The integration and interpretation of omics data necessitate sophisticated computational tools to provide meaningful insights, due to their complexity and scale. Besides improvements in this area and the available tools to integrate plant omics data, their analysis could still be a major bottleneck, mainly due to the overwhelmingamount of data. Recent reviews have proposed a methodological guideline for the plant integration of omics data [274,275]. Moreover, the application of artificial intelligence algorithms, particularly machine learning (ML) and deep learning, are promising approaches for deciphering complex omics data in plant defense research [276]. The first studies into multi-omics data integration by applying ML to predict the genes involved in SPM synthesis are being published [277].

## 7. Conclusions

Taken together, given the different strategies and approaches currently applied to study SPM, and other emerging technologies in proteomics that we highlight here, proteomics supports the analysis of the many unique properties of the proteome. The proteome is a highly structured entity in which proteins exert their function at a specific time and at a specific location, and in physical or functional association with other proteins or molecules. Therefore, the proteome is an interactively, spatially and temporally dynamic entity. It is evident that an integrative omics approach is one in which proteomics, combined with transcriptomics/genomics and metabolomics, will provide a less biased piece of information. Only proteomics can capture the spatial distribution of proteoforms and their abundance, as well as the interactions that occur between protein–protein or protein–metabolite. Improvement in modern MS-based proteomics technologies and the availability of plant sequence information will no longer be bottlenecks for the study of plant-specialized metabolism. Thus, proteomics in omics-driven studies will contribute to deciphering the many unknowns that still remain in plant-specialized metabolism research.

## Data Availability

Not applicable.
